# Limb-Enhancer Genie: An accessible resource of accurate enhancer predictions in the developing limb

**DOI:** 10.1371/journal.pcbi.1005720

**Published:** 2017-08-21

**Authors:** Remo Monti, Iros Barozzi, Marco Osterwalder, Elizabeth Lee, Momoe Kato, Tyler H. Garvin, Ingrid Plajzer-Frick, Catherine S. Pickle, Jennifer A. Akiyama, Veena Afzal, Niko Beerenwinkel, Diane E. Dickel, Axel Visel, Len A. Pennacchio

**Affiliations:** 1 Lawrence Berkeley National Laboratory, Berkeley, California, United States of America; 2 Joint Genome Institute, U.S. Department of Energy, Walnut Creek, California, United States of America; 3 Department of Biosystems Science and Engineering, ETH Zurich, Basel, Switzerland; 4 School of Natural Sciences, University of California, Merced, California, United States of America; Ottawa University, CANADA

## Abstract

Epigenomic mapping of enhancer-associated chromatin modifications facilitates the genome-wide discovery of tissue-specific enhancers *in vivo*. However, reliance on single chromatin marks leads to high rates of false-positive predictions. More sophisticated, integrative methods have been described, but commonly suffer from limited accessibility to the resulting predictions and reduced biological interpretability. Here we present the Limb-Enhancer Genie (LEG), a collection of highly accurate, genome-wide predictions of enhancers in the developing limb, available through a user-friendly online interface. We predict limb enhancers using a combination of >50 published limb-specific datasets and clusters of evolutionarily conserved transcription factor binding sites, taking advantage of the patterns observed at previously *in vivo* validated elements. By combining different statistical models, our approach outperforms current state-of-the-art methods and provides interpretable measures of feature importance. Our results indicate that including a previously unappreciated score that quantifies tissue-specific nuclease accessibility significantly improves prediction performance. We demonstrate the utility of our approach through *in vivo* validation of newly predicted elements. Moreover, we describe general features that can guide the type of datasets to include when predicting tissue-specific enhancers genome-wide, while providing an accessible resource to the general biological community and facilitating the functional interpretation of genetic studies of limb malformations.

## Author summary

The majority of the human genome does not code for proteins. Regulatory roles have been ascribed to a growing fraction of the non-coding genome. Enhancers, short stretches of non-coding DNA, confer spatial and temporal specificity to gene expression patterns. These regions are essential to the proper development of multi-cellular organisms and, when mutated, can give rise to congenital malformations and contribute to human disease. In line with these observations, a large fraction of the genetic variation associated to developmental abnormalities cannot be narrowed down to protein-coding defects, suggesting these variants actually reside in functional non-coding regions, such as enhancers. However, identification of enhancers in the mammalian genomes that are functional *in vivo* remains a difficult task. Here we combine chromatin and DNA-sequence data from the mouse genome in a machine learning framework resulting in the Limb-Enhancer Genie (LEG), an accurate and easily accessible collection of predicted enhancers active in the developing limbs. LEG outperforms state-of-the-art approaches, as testified by the high fraction of newly tested elements that we validated in the developing mouse embryo *in vivo*. To grant the community access to our predictions and their mappings to the human genome, we established a user-friendly web-interface.

## Introduction

Mammalian body plans are shaped by the precise spatiotemporal execution of transcriptional programs [1], which have been shown to rely on the coordinated activity of enhancers [2]. Despite the increased availability of epigenomic data, the identification of these short, *cis*-regulatory DNA sequences in the vast non-coding portions of mammalian genomes has proven to be a difficult task. Indirect measurements suggest that hundreds of thousands of enhancers populate mammalian genomes [3], but only a few thousand of them have been validated for their activity *in vivo* [4]. A wide range of experimental and computational approaches have been applied to the prediction of regions showing enhancer activity *in vivo*, including: 1) Evolutionary conservation [5]; 2) Chromatin signatures, such as the binding of the co-activator p300 [6] or the acetylation of lysine residue 27 of histone H3 (H3K27ac) [7,8]; 3) Chromatin accessibility to DNase I digestion [9]; 4) Genomic sequence signatures, such as the presence of binding sites for relevant transcription factors (TFs) [10]; 5) Combinations of the former strategies. Despite significant advancements in enhancer identification, through the generation of genome-wide datasets and their integration using supervised [11–15] or unsupervised [16,17] models, all available approaches to date suffer from one or more of the following limitations: 1) Lack of integration of chromatin and sequence features that are immediately relevant to the tissue(s) and the developmental stage(s) under consideration; 2) Lack of thorough, biological interpretation of the features driving the prediction, which in turn is a key requirement to instruct experiments and more refined models in the future; 3) Lack of appropriate negative controls–e.g. the use of random genomic intervals instead of regions showing (at least partially) a known signature of enhancers but failing to display tissue-specific activities when tested *in vivo*; 4) Lack of user-friendly access to the *de novo* predictions, limiting the value of the resulting resources for the community of experimental as well as computational biologists.

In this work we integrate multiple machine learning approaches in order to produce robust predictions of enhancers active in the developing limbs of mouse embryos at embryonic day 11.5 (E11.5). By focusing on this well-studied developmental system [18,19], we are able to overcome the limitations described above and outperform previously described state-of-the-art approaches [11,12]. First, we exclusively considered datasets generated from embryonic limbs (with one exception, a DNase I hypersensitivity dataset from headless embryos) at the relevant developmental time points (E10.5, E11.5 and E12.5), including the binding profiles for CTCF, the cohesin complex, and a large panel of histone modifications [3,6,8,20,21]. Among the latter we also included recently published ChIP-seq data from specific limb compartments [22]. Importantly, we trained statistical models that provide intrinsically interpretable measures of feature importance (LASSO and random forests). This allowed us to identify a previously unreported feature capable of significantly improving predictions, namely limb-specific DNase I enrichment. The predictive power of this feature was confirmed using data from other tissues (central nervous system and facial prominence) at the same developmental time point. We additionally trained models based on clusters of evolutionary conserved binding sites for those TFs expressed in the developing limbs, and formally integrated these results with the chromatin features described above. We used a set of >200 validated limb-enhancers and ~2,000 negatively tested regions corresponding to either validated elements active in tissues other than limb or that were previously selected based on chromatin or sequence features of active enhancers but failed validation due to absence of reproducible reporter activity [4]. Based on our results, we were able to confirm the *in vivo* activity of three out of four newly predicted enhancers in the vicinity of the *Hand2* gene, an important regulator of limb morphogenesis [23,24].

Importantly, our genome-wide predictions can be queried through a user-friendly web interface named LEG (Limb-Enhancer Genie), which is available at http://leg.lbl.gov/. Since a large fraction of limb developmental enhancers are evolutionarily conserved between human and mouse [6], the user can also input regions from the human genome. The complete set of predictions along with all the sequencing datasets re-analyzed in this study are available for browsing via a public track hub (see Methods) on the UCSC genome browser [25].

By providing the community with significantly improved genome-wide maps of the enhancer landscape underlying limb development, our results will assist the functional interpretation of genetic studies assessing human developmental diseases. Moreover, the analysis of the feature importance in the trained models provides novel generalizable insights into the chromatin signature of developmental enhancers that will help guide the design of predictive models in tissues other than limb.

## Results

**Fig 1:**
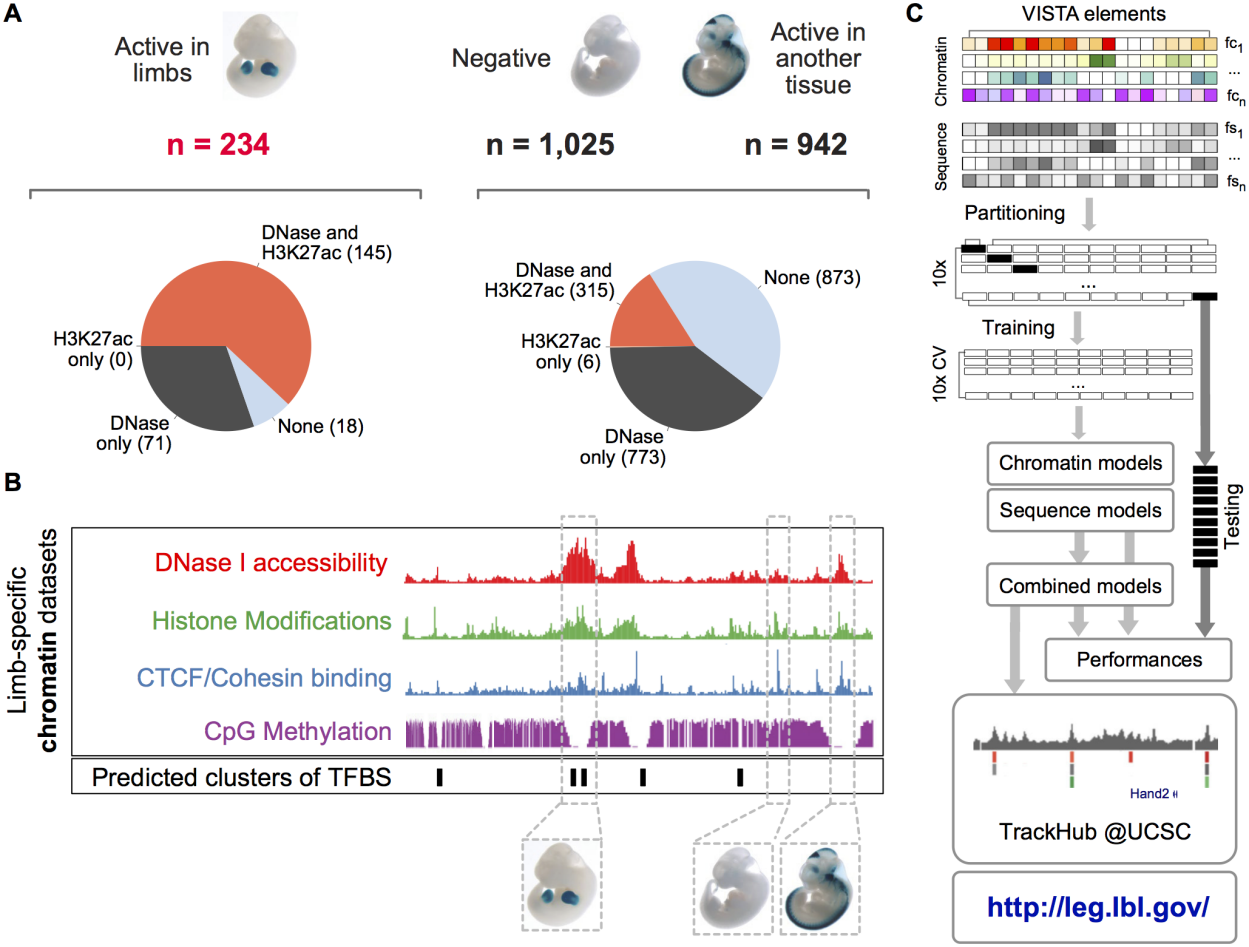
Overview of the approach. (**A**) *In vivo* tested sequences from VISTA [4] considered in this study. For both limb-enhancers (left) as well as sequences not active in the developing limbs (right) the overlap with H3K27ac peaks and/or DNase I hypersensitive sites is shown as pie charts. (**B**) Schematic of the different classes of chromatin and sequence features considered in this study. (**C**) Summary of the machine learning strategy. After calculation of the relevant chromatin and sequence features for all observations, the data was partitioned into ten equally sized bins, retaining the original ratio of positive to negative observations. Training was performed using 10-fold cross-validation (CV), separately for each model (LASSO, RF, SVM) and categories of features (chromatin, sequence). The performances of these models as well as their combinations were evaluated on the ten independent, non-overlapping test sets. Models were then trained using the entire set of observations, and genome-wide predictions were made available through a track hub (see Methods) for the UCSC genome browser [25] and through a user-friendly web interface at http://leg.lbl.gov/.

DNase I accessibility and H3K27ac are routinely used to identify tissue-specific putative enhancers in the human and the mouse genomes [7,8,26,27]. We first aimed to determine the sensitivity and specificity of these marks, using the developing limb as a test case (Fig 1A). Even when used in combination, these marks suffer from both high false positive and false negative rates. More than 50% (1,094/1,967) of the limb-negative elements in the VISTA collection [4] overlapped H3K27ac-enriched or DNase I accessible regions (false positives). At the same time, a fraction of enhancers truly active in the limbs at E11.5 (18/234) were still missed by both assays (false negatives). These results prompted us to set up a more integrative approach towards more effective limb enhancer discovery.

To this aim, we took advantage of >50 recently published limb-specific, genome-wide datasets (S2 Table) from four major categories of chromatin features (namely DNase I accessibility, six histone modification and co-activator p300, CpG methylation, and the binding of CTCF/cohesin, Fig 1B). We chose to use the limb as a model based on the extensive available chromatin data [3,8,20,21], which includes robust time series spanning three closely spaced developmental time points (E10.5, E11.5 and E12.5) and hundreds of *in vivo* validated elements [4]. These datasets comprise two subregion-specific sets (representative of two important signaling centers in the developing limb, namely the Apical Ectodermal Ridge, or AER, and the Zone of Polarizing Activity, or ZPA, [22]). Importantly, including DNase I digestion patterns from whole embryos whose heads had been removed [28] allowed the estimation of tissue-specific DNase I enrichment scores. In addition to the chromatin state, we also incorporated one class of sequence features, i.e. the predicted clusters of evolutionary conserved binding sites for those TFs expressed in the developing limbs.

In order to better understand the relative contribution of chromatin and sequence features, our strategy first considered them separately, and then in combination (Fig 1C). To improve robustness, we partitioned our set of 234 limb enhancers (positive examples) and 1,967 regions negative for activity in the limbs (1,025 negatively tested regions and 942 showing activity in another tissue, Fig 1A and S1 Table) into ten equally sized bins with constant ratio of positive to negative observations (where one bin was used in turn as a test set, whereas the remaining nine constituted the training set). The model parameters were learnt using 10-fold cross-validation over the training set. The models trained include least absolute shrinkage and selection operator (LASSO, [29]), support vector machines (SVM, [30]) and random forests (RF, [31]). Model performances were compared across the ten independent test sets. Predictions from different models and distinct sets of features were then combined using ridge regression or a weighted sum of ranks approach (SOR). For the final prediction of enhancers genome-wide, the training step was re-iterated using the entire dataset, and the resulting models and their combinations were used to call enhancer regions using a sliding window (see Methods).

LASSO and RF allow evaluation of the importance of each feature for model performance. This enabled us to gain insights into the biological relevance of the most predictive features. A notable novelty in our method is the use of a previously unappreciated feature, a score measuring increased tissue-specific DNase I accessibility (DNase I enrichment), into our predictive models. Unexpectedly, we found the headless embryo DNase I accessibility pattern to be well correlated with the DNase I specific for fore- and hindlimbs (*r* = 0.87 and 0.85, respectively, within the VISTA-dataset). This prompted us to hypothesize that including the ratio between the DNase I signal from limbs and the headless embryos would better capture the limb-specific changes in DNase I accessibility, as compared to the limb DNase I signal alone. Unsupervised clustering of the training set followed by visual inspection of the results provided additional evidence to support this hypothesis and revealed further interesting groups (S1 Fig). Next to clusters of negative elements showing predominantly low values across all features, the set contained groups of negatively tested elements showing known chromatin signatures associated with regulatory elements other than enhancers. One group contained mostly promoter-like elements (high H3K4me3 and H3K9ac), while others resembled insulators (high CTCF and Smc1a) or Polycomb-associated heterochromatin (high H3K27me3) [32,33]. These qualitative observations prompted us to include these chromatin features, along with the DNase I enrichment score as described above, into our machine learning strategy.

**Fig 2:**
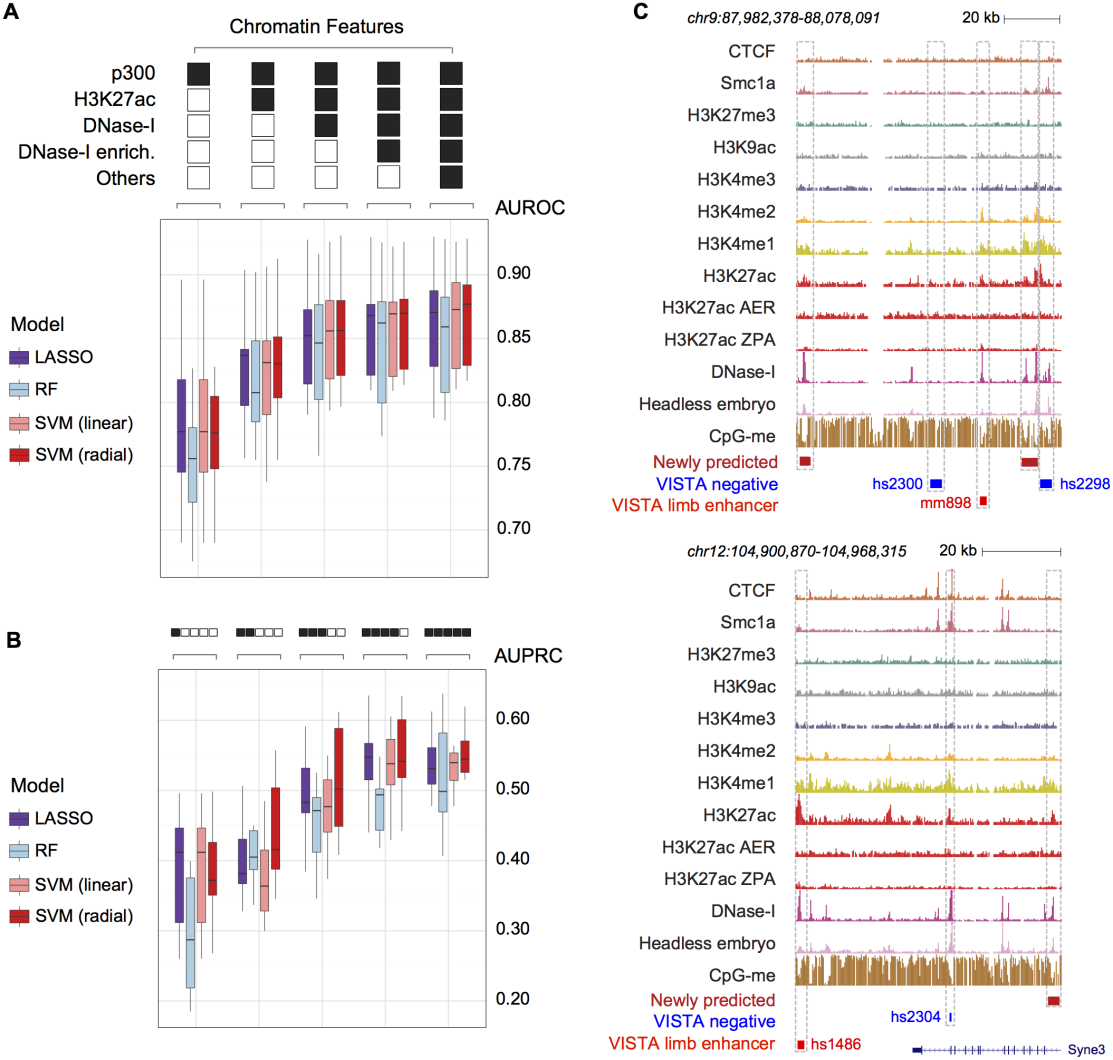
Limb-specific chromatin features accurately predict limb-enhancers. (**A**) Box plots showing the AUROC estimated on the ten leave-one-out test sets, considering an increasingly larger set of chromatin features (left to right, outliers not shown). (**B**) Same as (A) but showing the AUPRC. (**C**) UCSC genome browser snapshots indicating two representative loci. Validated limb enhancers (bright red elements) showed different features than nearby regions that tested negative *in vivo* (blue). In particular, they displayed a higher DNase I enrichment (compare *DNase I* to *Headless embryo*). *De novo* limb-enhancers predicted based on combined models are also shown (dark red).

We first built models of increasing complexity, starting from p300 alone and incrementally adding H3K27ac, DNase I, DNase I enrichment and all the remaining chromatin features (Fig 2A and 2B). The median AUROC (Area Under the Receiver Operating characteristic Curve) as well as the median AUPRC (Area Under the Precision Recall Curve) steadily increased by including more features (as assessed on the independent test sets). This was observed consistently across the different models. Considering the one showing the highest performances (namely the radial SVM), the median AUPRC when training only on p300 was 0.372, a figure that increased to 0.412 when including H3K27ac, to 0.502 if considering also DNase I, and finally to 0.542 and 0.545 if adding the DNase I enrichment or all the features, respectively (Fig 2B, S3 and S5 Tables). Interestingly, models trained only on H3K27ac/p300 and DNase I (including the enrichment over the head-less embryo samples) reached a performance almost as high as the full set of chromatin features on the VISTA dataset. However, these additional features are well-known marks for categories of regulatory elements–e.g. insulators and promoters–that are under-represented in our training set but are widespread genome-wide. In fact, by overlapping the CTCF-bound sites in the developing limbs (which are enriched for insulator sequences) [21] with the *de novo* enhancer predictions genome-wide using different feature subsets, the effect of including the additional features became more evident. While models trained on H3K27ac/p300 and DNase I alone showed 10 to 20% overlap with CTCF-bound sites across a wide range of predicted values, models trained on the complete set of features showed less than 5% (S2 Fig), potentially removing a fraction of false positive predictions.

**Fig 3:**
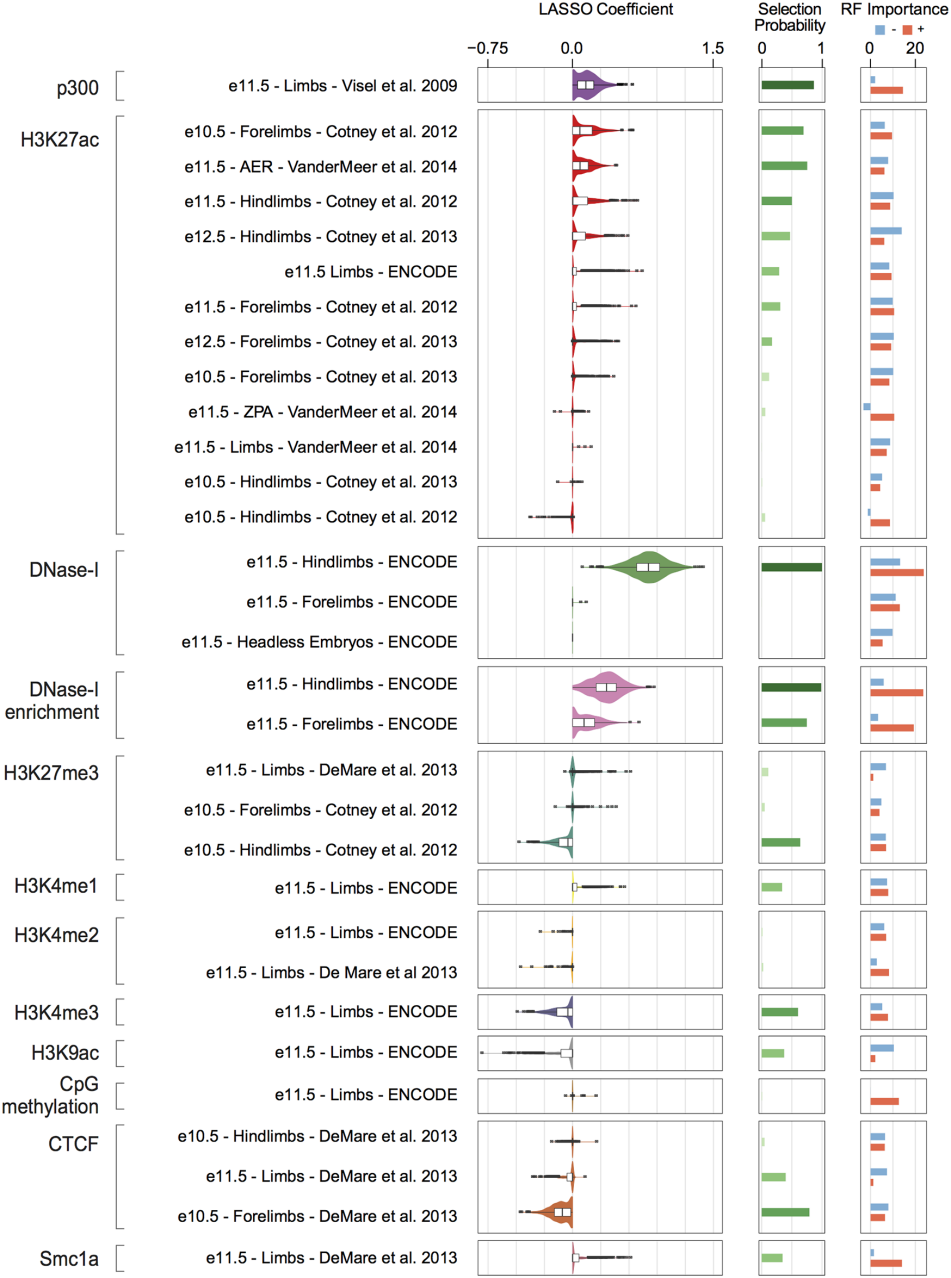
Biological interpretation of the chromatin models based on feature importance. For each macro-category (on the far left), each dataset considered is indicated (stage, structure and reference publication are specified), followed by three distinct plots showing (left to right): (1) a box plot overlaid to a violin plot showing the distribution of the coefficients assigned to each particular feature by LASSO; (2) the selection probability as estimated by *Bootstrap LASSO* (darker shades of green indicating higher probability); (3) the feature importance estimated as mean decrease in accuracy by RF (separately for the positive and the negative classes, indicated by red and light blue bars, respectively).

**Fig 4:**
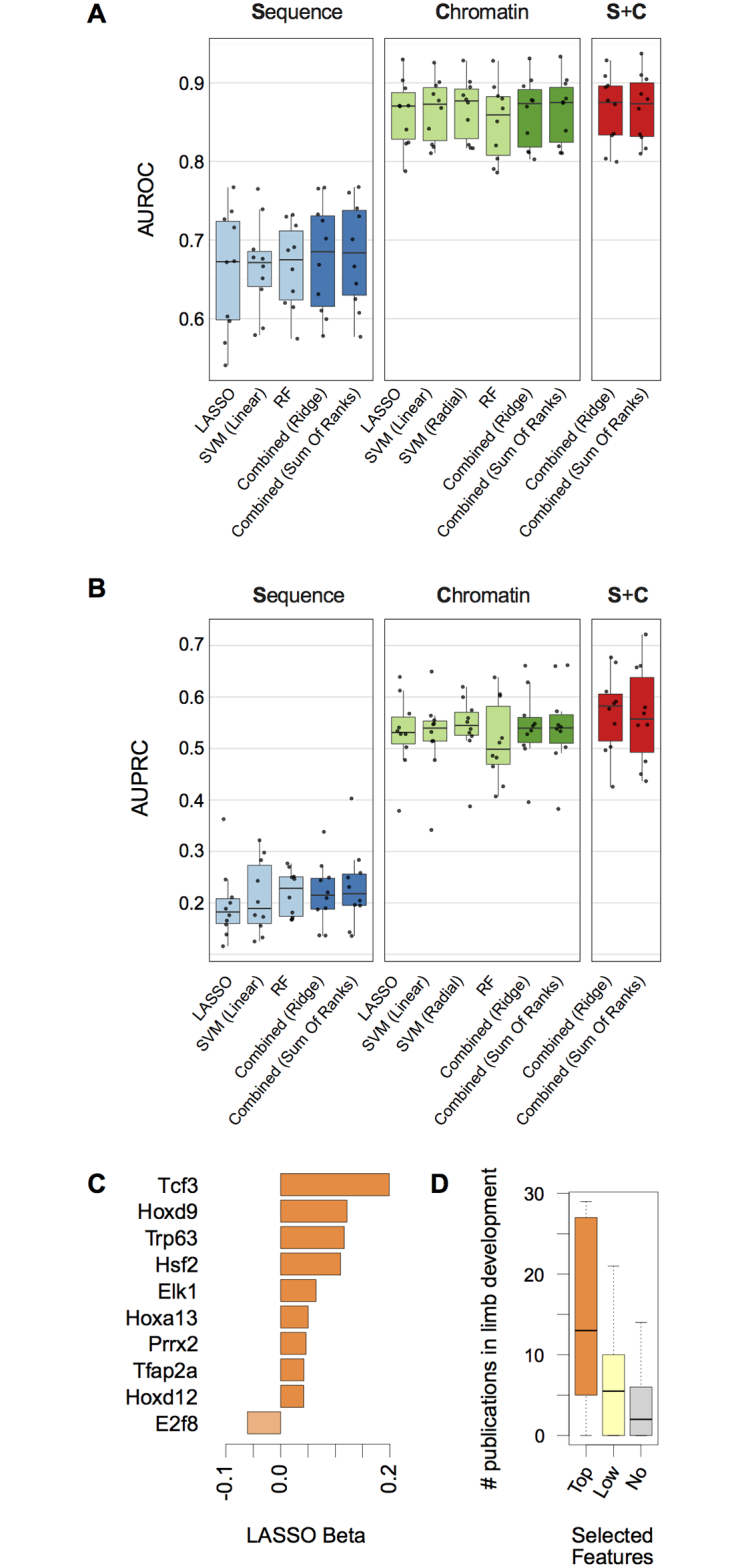
Modeling limb enhancers sequence composition and its integration with chromatin information. (**A**) AUROC estimated on the ten leave-one-out test sets for all the indicated models (Sequence, Chromatin or their combination, S+C). (**B**) Same as (A) but showing AUPRC. (**C**) Bar plot showing the average coefficient from the LASSO models for those sequence features selected in 9/10 of splits during LASSO training and reported in the top 25% in terms of mean decrease in accuracy, as estimated during RF training. (**D**) Boxplot showing the distribution of the total number of publications in limb development for the genes shown in (C) (*Top*, orange) as well as for those TFs whose corresponding features were selected in at least one CV-fold (but less than nine, *Low*, light yellow) or in none (*No*, grey) (outliers not shown).

A qualitative evaluation of complex loci (Fig 2C) indicated that validated limb enhancers show a higher DNase I enrichment (*DNase I* versus *Headless embryo*) as compared to nearby regions that tested negative *in vivo*. Given these observations, we then sought to systematically and quantitatively assess the relative importance of the different chromatin features. Specifically, the estimated coefficients from the LASSO and the mean decrease in accuracy estimated by the RF were evaluated. We also estimated a selection probability for each predictor by *Bootstrap LASSO* (see Methods). The results are summarized in Fig 3 and S9 Table. DNase I accessibility as well as the limb-specific DNase I enrichment were systematically co-selected (>0.98 selection probability by *Bootstrap LASSO*) and showed the highest coefficients as well as importance in the RF. Co-selection of these two features further supports a substantial rather than incremental role of tissue-specific DNase I enrichment in identifying active enhancers *in vivo*. Interestingly, while both the DNase I enrichment values from hind- and forelimbs were often selected and assigned positive coefficients, the forelimb DNase I signal was only selected in 2 out of 1,000 bootstrap samples, in contrast to the the hindlimb DNase I signal which was selected every time. We re-ran the *Bootstrap LASSO* after removing the hindlimb DNase I and found that the DNase I from forelimbs was selected with a probability of one and almost identical performances. This indicates that the dataset from hindlimb might be favored for technical rather than biological reasons. Other features that were selected with high probabilities, but less often than DNase I associated features, were p300, CTCF, H3K27ac, H3K27me3 and H3K4me3 (all showing a selection probability >0.6). The small size of the training set combined with the multiplicity of classes of regulatory elements, each represented only by a few examples (S1 Fig), was likely responsible for the lower selection probabilities of these features. As expected, p300 and bulk H3K27ac are the most important predictors after DNA accessibility (selection probability >0.75). On the other hand, when we assessed the contribution of the H3K27ac datasets specific for different sub regions of the limb, we found that the ZPA-specific profile was very unlikely to be selected, in contrast to the AER-specific one, which was often included in the LASSO models with a positive coefficient. This region-specific feature is selected >75% of the time, often together with H3K27ac profiles from whole limbs. Thus, our findings highlight the importance of gathering chromatin information at a finer scale in order to be able to identify enhancers with more sub-regional-specific activity. The histone modification H3K4me3 (and to a lesser extent H3K9ac, which are both usually found at promoter regions [33]), was assigned a negative coefficient, as was the mark H3K27me3, which has been associated with inactive, poised enhancers [34] or more generally with Polycomb-associated heterochromatin. The two proteins CTCF and Smc1a, while often co-bound to DNA on the same genomic elements (mainly insulators or promoters) [32], are assigned coefficients of opposite sign (negative for CTCF, positive for Smc1a), indicating that cohesin but not CTCF is more generally associated with enhancer function. Smc1a was assigned a high importance for the prediction of limb-enhancers by the RF, while it was selected only in 35% of the bootstrap-samples by LASSO. CpG methylation was also found to be rather important in the RF predictions, but very unlikely to be included in the LASSO models. A possible explanation could be the implicit accounting for feature interactions in the RF, which remain unappreciated by the LASSO. Nevertheless, the small size of the training set impinged our ability to explicitly tease out these combinatorial relationships. Taken together, these results are in line with the expectation that different models are able to capture distinct aspects of the data. This prompted us to combine the results from the multiple models into a single, unified predictive score. Two different approaches were applied to this end: ridge regression (i.e. finding optimal weights to combine the outputs from the single classifiers) and an approach based on the weighted sum of the individual output-ranks. These strategies led to a significant improvement in the AUPRC as compared to RF (*p*-value = 0.03, one-tailed Wilcoxon signed-rank test, Fig 4B) and a smaller improvement when considering LASSO or linear SVM (*p* = 0.05, combined ridge model), but it was not significant as compared to radial SVM (*p* = 0.57, combined ridge model).

We then asked whether considering clusters of evolutionary conserved TF-binding sites could lead to a more consistent increase in the predictive power of the combined models. In order to limit the number of input features, we only considered binding motifs for TFs expressed in the developing limbs (see Methods, S3 and S4 Tables). The overall performances of the sequence features alone were markedly lower than those achieved by chromatin (Fig 4A and 4B, S3, S4 and S5 Tables). Even the application of gkm-SVM [35,36], a motif-agnostic machine learning approach which has been previously applied to enhancer prediction [37], lead to performances comparable to those achieved by our models (median AUPRC of 0.197, S11 Table and Discussion). However, combining our sequence- and chromatin-based predictions using ridge regression significantly outperformed the combined chromatin model (Figs 4B, S3 and S4; *p* = 0.019 in terms of AUPRC, one-tailed Wilcoxon signed-rank test; *p* = 0.08 when considering the combined SOR) and the best single chromatin model (namely the radial SVM, *p* = 0.024, one-tailed Wilcoxon signed-rank test). Model performances across the test sets are reported in S5 Table. Similar to the chromatin features, we combined the importance measurements from the LASSO and the RF, and short-listed the most relevant TFs (Fig 4C and S10 Table). Interestingly, these TFs are over-represented in publications in the field of limb development as compared to TFs whose motifs were selected less frequently or not selected at all (Fig 4D, *p*-value = 0.02, Mann-Whitney test, *Top* vs *No*). Among the most frequently selected motifs are those of *Hoxa13* and *Hoxd9*, which are known regulators of digits and stylopod development, respectively [38]. *Tp63* is a critical factor for epithelial development that, when mutated, can lead to severe developmental defects including complete absence of limbs [39]. *Tfap2a* has been previously associated with distal outgrowth of the developing limbs [40]. While *Tp63* and *Tfap2a* have been associated to limb development, these results suggest they might exert their function by binding to enhancer elements. These findings underscore the importance of applying interpretable machine learning approaches to highlight relevant features, in turn helping to formulate new experimental hypotheses.

**Fig 5:**
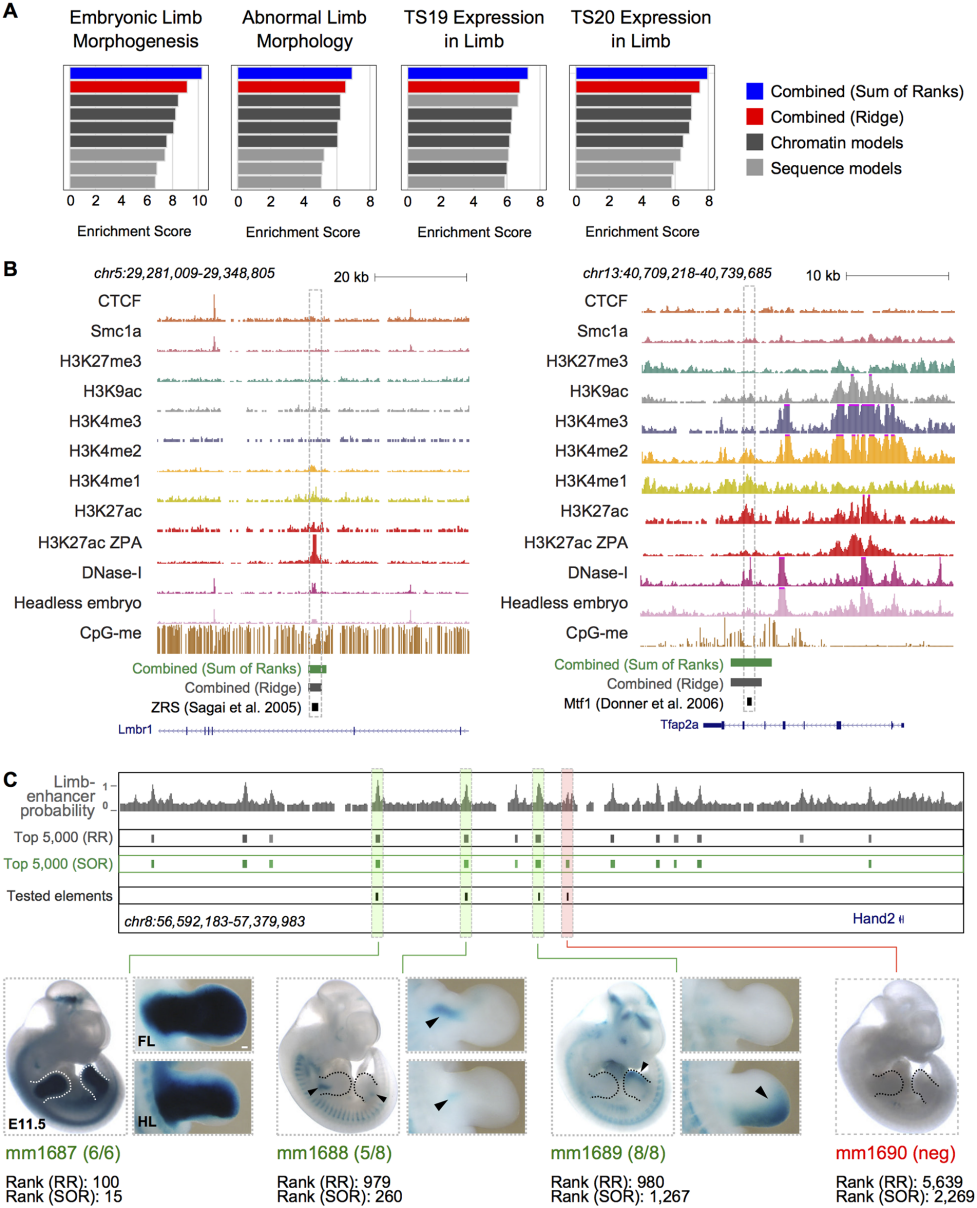
LEG predicts *bona fide* limb-enhancers genome-wide. (**A**) Overall enrichment scores (see Methods) for the indicated functional terms based on the proximity of the newly predicted elements to the genes annotated within each category (see also S6 Fig). (**B**) UCSC genome browser [25] snapshots showing the landscape at two previously *in vivo* validated limb-enhancers that were not part of the training set but were identified in the top 10,000 predictions. The region on the left is the ZRS (ZPA Regulatory Sequence), a known regulatory element for *Shh* [43]; the one on the right is an intronic enhancer of *Tfap2a* [44]. (**C**) UCSC genome browser snapshot of the *Hand2* gene locus. The probability of being a limb-enhancer (Ridge model) along with the top 5,000 predictions from both the Ridge Regression (RR) and the Sum Of Ranks (SOR) combined models are shown. The four elements tested for activity in the developing limbs are highlighted in boxes (green for those showing activity in the limbs at E11.5, red if negative). *LacZ* reporter staining (blue) indicates enhancer activities in the fore- and hindlimb mesenchyme at E11.5. One representative whole mount picture is reported for each tested element. Pictures of a representative forelimb and hindlimb are provided for the validated enhancers. Reproducibility is indicated in brackets below each whole mount picture, along with the corresponding VISTA identifier. The ranks for both combined scores (RR, SOR) are also reported. Scale bar, 100 μm.

By combining the predictions from both the chromatin- and sequence-based models, our strategy outperformed the state-of-the-art approaches [11,12] both in terms of AUPRC and AUROC (Table 1). In line with this, the distributions of predicted values between the positive and negative regions in the training set showed a stronger separation in our combined models as compared to EnhancerFinder and EMERGE (Kolmogorov–Smirnov statistic, S5 Fig). We also compared the performance of our combined models to the predictive power of the strong enhancers chromatin states defined using two ChromHMM models [17], trained using eight histone modifications from two distinct biological replicates [41] from E11.5 limbs (see Extended Methods). This resulted in a recall of 0.162 and a precision of 0.447 for the enhancer calls from one of the two replicates. At the same level of recall, our combined models reached a much higher precision (0.787 and 0.741 for the ridge regression and the SOR approach, respectively). The second replicate led to comparable conclusions (recall of 0.256 and precision of 0.417 for the ChromHMM calls, as compared to a precision of 0.732 and 0.710, given that level of recall in our combined models). These results prompted us to train the models using the complete set of observations and to run them genome-wide. The mouse genome was tiled into overlapping windows of 2kb, which were assigned prediction values for tissue-specific enhancer activity *in vivo* using all models. The resulting predictions for each single model (either LASSO, SVM or RF) and type of feature considered (chromatin, sequence) as well as their combination were ranked, and the 20,000 highest scoring regions were binned into 10 groups (see Methods and S8, S13 and S14 Tables). These were used to evaluate the enrichment for proximity to genes involved in limb development and expressed in Theiler stages 19 and 20 (corresponding to the window from E11 to E13) using GREAT [42] (Figs 5A and S6). The enrichments from the combined predictions were higher than those of any single model trained on chromatin or clusters of conserved TF-binding sites alone, indicating that the combined models can identify thousands of *bona fide*, previously uncharacterized, enhancers. Interestingly, while showing lower performances on the test sets as compared to the combined ridge classifier (Fig 4B), the SOR showed the highest enrichment in terms of proximity to genes relevant to limb development (Fig 5A), especially for the highest-ranking elements (S6 Fig).

**Table 1:** LEG performances compared to two state-of-the-art approaches.

Method	AUROC	AUPRC
LEG (combined, ridge)	*0*.*875*	*0*.*582*
EnhancerFinder [11]	0.828	0.453
EMERGE [12]	0.820	0.327

Average performances were determined by cross-validation across the same test/training partitions used for all models (Fig 1C).

To further corroborate our predictions, we searched the literature for developmental limb enhancers that were robustly validated *in vivo* but are not part of the VISTA collection. We identified five elements, all of which overlapped the 10,000 highest scoring predictions (considering either the ridge regression or the sum of ranks, S12 Table). One of these regions is the ZRS (ZPA Regulatory Sequence), which is a well-known enhancer controlling the expression of *Shh* in the ZPA [43]. This element consistently ranked very high across both the sequence- and chromatin-based predictions, in line with the abundance of conserved TF-binding sites and the presence of a strong limb-specific DNase I signal (Fig 5B, left panel, and S12 Table). A further, independently identified element that drives the expression of *Tfap2a* in limbs and face [44] was correctly predicted within an intron of the *Tfap2a* gene itself (Fig 5B, right panel).

We then additionally verified the ability of the proposed approach to identify *bona fide* limb-enhancers by choosing four newly predicted elements close to the developmental regulator TF *Hand2* and testing them *in vivo* through mouse transgenic enhancer-LacZ reporter assays (Fig 5C). Recently published promoter-Capture-C data [45] from developing limbs at E11.5 revealed that these elements are indeed located in a domain contacting the *Hand2* promoter with high frequency (2/4 reaching statistical significance, S7 Fig) demonstrating their potential to act as enhancers for this gene. *Hand2* displays critical developmental functions in various embryonic tissues such as the limb [23,24], the heart [46–48] and the craniofacial structures [49]. However, the *Hand2* limb-specific enhancer landscape has been poorly characterized so far. Three out of four tested elements displayed reproducible *LacZ* reporter staining at E11.5, with patterns of activity specific to limbs and overlapping well-known subdomains of *Hand2* expression [50]. Interestingly, the only element that tested negative also showed the lowest predicted combined score (Fig 5C and S15 Table).

Finally, we made the genome-wide predictions available at http://leg.lbl.gov/. These can be directly and systematically queried through a user-friendly interface. The website also provides two tutorials that leverage published datasets that were not used in the predictions.

## Discussion

In this work, we integrated >50 genome-wide chromatin datasets with sequence information and were able to improve our ability to recognize limb enhancers over previously published approaches. These include EnhancerFinder [11] and EMERGE [12] which represent computational state-of-the-art tools in the field (Table 1) and to our knowledge are the only two studies that employed the VISTA dataset in a way that is comparable to our approach. Combined with making the predictions readily available via a user-friendly interface, another advantage of the presented study is the extensive application of machine learning models (LASSO, RF) that are intrinsically designed to provide feature importance. In this way, we have shown that including a limb-specific DNase I enrichment score dramatically improves the prediction of developmental limb-enhancers in terms of both precision and recall over incorporating just the commonly used histone-mark H3K27ac (Fig 2). The availability of both DNase I accessibility and H3K27ac ChIP-seq data for midbrain, hindbrain, neural tube and facial prominence tissues at E11.5 (S17 Table) allowed us to reproduce this finding also in tissues other than limb at the same developmental time point. To this aim, we fit logistic regression models including p300, H3K27ac, DNase I and DNase I enrichment features consecutively, and found that inclusion of the DNase I enrichment scores on top of the other features considerably and significantly improved the performance of all the neuronal tissues and to a lesser extent of facial prominence (S8 Fig). Our ability to evaluate the performances for craniofacial enhancers is affected by the lower number of validated examples (74 versus 274, 310 and 196 for hindbrain, midbrain and neural tube, respectively) leading to greater variance in the overall performance. In addition to the DNase I enrichment score, the chromatin features H3K4me3, H3K9ac, H3K27me3 as well as the binding of CTCF and Smc1a were selected as informative (Fig 3). The inclusion of features other than H3K27ac and DNase I accessibility reflects the presence of VISTA elements showing combinations of these genomic features in the developing limbs, including many that failed to validate *in vivo*. These are very likely to be insulators, unannotated canonical promoters or poised enhancers, rather than active enhancers (S1 Fig). As many regions in VISTA were selected specifically because they showed canonical enhancer marks, these other classes are likely under-represented in our training data. Nevertheless, these types of elements could be misclassified as enhancers when only DNase I and H3K27ac are used for genome-wide scanning, and our approach proved effective at exploiting this information. When moving to the scale of genome-wide prediction, the small but significant improvements in performance observed when including all these features lead to important differences in the types of elements that are predicted. For example, models trained on H3K27ac and DNase I only are more enriched for insulator-like elements, as indicated by a larger overlap with CTCF-bound regions (S2 Fig).

Of note, LASSO also systematically selected with a positive coefficient the H3K27ac dataset specific for the AER sub region (Fig 3). This demonstrates the value of sub-regional-specific datasets. Nevertheless, there are two issues that need to be addressed in order to fully harness this kind of datasets in the future. First, the current number of tested VISTA enhancers (and more in general in the literature) showing activity for each different sub-region is still low. Second of all, there are very few high-quality genomic datasets generated from sub-regional dissected tissues available at present.

The performances of the sequence-based models were on the other hand lower than what we observed when incorporating experimentally derived, chromatin data (Fig 4). The performance of our models are seemingly lower than previously reported [37,51]. Previous publications mainly focused on the prediction of sequences with enhancer activity using randomly selected genomic regions (matched by GC- and repeat- content) as negative examples. In this study, we focused on a different problem, i.e. the identification of enhancers showing limb-specific activity, against regions that either show enhancer activity in a different tissue than limb, or anyway were selected based on partial experimental evidence of activity, but failed *in vivo* validation. In fact, when we applied gkm-SVM [35,36], a machine learning approach previously applied to enhancer prediction [37], we observed performances comparable to those achieved by our sequence-based models (S11 Table). More in general, the identification of transcription factor binding sites genome-wide suffers from a high false positive rate, a problem that was only partially mitigated by leveraging the information of TF-binding-sites-clustering and the evolutionary conservation of these sites. On top of this, developing tissues are complex, heterogeneous mixtures of lineages giving rise to multiple cell-types, each one of which depends on only partially overlapping gene regulatory networks. As such, the diversity of regulatory elements at the sequence level is expected to be much greater in tissues than in more homogeneous, *in vitro* cell populations. This factor is likely to have a major impact on the signal-to-noise ratio for the identification of sequence-encoded features. In line with this, the most important TFs identified by the sequence-based models are enriched for general regulators involved in enhancer function in the limb, like the *Hox* family genes, *Tp63* or *Tfap2a* (Fig 4C and 4D). Despite these limitations, we found multiple evidences supporting the value of integrating both chromatin and sequence features in our predictive framework. These included functional analysis of the *de novo* predictions using GREAT [42] (Figs 5A and S4), our *in vivo* validation of three out of four newly predicted enhancers very likely involved in the transcriptional regulation of *Hand2* (Fig 5C and S15 Table), and the recapture of previously validated limb-enhancers from a number of independent studies (Fig 5B and S12 Table). Of note, even though the incorporation of the sequence features significantly improved the predictions *per se* (Fig 4A and 4B), the use of evolutionary conserved TF-binding sites still led to a considerable number of false positive (S9 Fig). A more unbiased approach to mitigate all the issues highlighted in this paragraph would be the generation of high-quality ChIP-seq profiles for the cell-type-specific TFs involved in the development of the embryonic tissue under study.

Overall, our results will help instruct future strategies for the identification of enhancers. Our analysis strongly suggests that the use of a limited number of features relevant to the developing organ system under scrutiny (chromatin accessibility, high enrichment for H3K27ac and p300 binding and low to no enrichment for H3K27me3, H3K4me3 and CTCF, see Fig 3), as well as the integration of a previously unappreciated feature, the DNase I enrichment, will likely improve the prediction of enhancers active across development and showing diverse tissue and sub-regional specificity. We expect this to be the case in the near future, as soon as the relevant genome-wide datasets are generated. We envision that measuring relative chromatin accessibility across tissues by means of ATAC-seq [52] might provide the same information (and in turn the same boost in predicting *bona fide* enhancers) as the DNase I enrichment score proposed here. At the same time, while more sophisticated computational models could be applied [53], these are currently limited by the size of the training set. Data gathering remains the major limiting step (e.g. validation in transgenic mouse lines is still relatively low-throughput). Technological advancements to increase the throughput as well as to standardize the assays (e.g. by site-specific integration of the reporter transgene in the genome) will soon be required and extremely beneficial. Importantly, by providing the community with an easy access to significantly improved genome-wide prediction maps of the enhancers active during limb development, we anticipate these results to be of value for both developmental biologists and human geneticists. Our web-interface (http://leg.lbl.gov/) can be queried using human genomic regions. This will specifically help the functional contextualization of human non-coding variants, pinpointing their contribution to limb malformations. As an example, the LEG predictions overlapping published H3K27ac-enriched regions in embryonic human limbs [20] (S16 Table and Extended Methods) are provided.

## Materials and methods

### Ethics statement

All animal work was reviewed and approved by the Lawrence Berkeley National Laboratory Animal Welfare Committee. All mice used in this study were housed at The Animal Care Facility (ACF) at LBNL. Mice were monitored daily for food and water intake, and inspected weekly by the Chair of the Animal Welfare and Research Committee and the head of the animal facility in consultation with the veterinary staff. The LBNL ACF is accredited by the American Association for the Accreditation of Laboratory Animal Care International (AAALAC, IACUC-approved animal protocol #290008).

### Validated enhancers from the VISTA enhancer browser

Human and murine validated elements were downloaded from the VISTA enhancer browser (http://enhancer.lbl.gov) [4] and mapped to mm10 coordinates using liftOver [25]. After filtering (see Extended Methods), 2,201 elements were used for machine learning (S1 Table).

### Data collection and analysis

ChIP-seq, DNase-seq, RNA-seq and CpG-methylation profiles collected for this study are listed in S2 Table. ChIP-seq and DNase I hypersensitivity reads were aligned to the mm10 release of the mouse genome (Dec. 2011, GRCm38) using bowtie2 [54]. ChIP-seq peaks were called using MACS v1.4.2 [55] for analysis regarding overlaps to enriched regions (not machine learning). RNA-seq datasets were aligned to the reference transcriptome (mm10, Ensembl 81 gene annotation release, [56]) using STAR v2.4.2a [57]. Transcripts were quantified with Stringtie v1.0.4 [58]. CpG-methylation bigWig tracks at base-pair resolution were downloaded from the ENCODE repository (http://www.encodeproject.org/) [3].

### Calculation of chromatin feature enrichments over genomic regions

Log2-RPKM quantifications for ChIP-seq and DNase I samples for each one of the 2,201 mm10-mapped VISTA elements were performed after expanding them to a minimum size of 2kb around their center. For ChIP-seq samples, enrichments were computed relative to the corresponding control samples (input DNA) (see Extended Methods). Scaling of the input features was performed as *z*-scores. For CpG-methylation, the average fraction of methylated CpGs was determined for each region.

### Estimation of clusters of TF-binding sites for limb-expressed TFs

Position weight matrices (PWMs) [59] (S3 and S4 Tables) were limited to those representing binding preferences of TFs potentially expressed in the developing limb (see Extended Methods). Putative TF-binding sites were identified using FIMO v4.10.2 [60], with a *p*-value cutoff of 10^−4^ and using GC-content matched backgrounds (see Extended Methods). Clusters were identified using a sliding window (500bp); binding sites were weighted by evolutionary sequence conservation, as estimated by *phastcons* [61]. Either the mouse or the human sequence was scanned according to which version was tested *in vivo* (S1 Table). A complete table of the scores for each TF-gene across the 2,201 VISTA elements is provided in S7 Table.

### Training the models and performance assessment

The observations were split into ten equally sized groups. Each group was used as test set exactly once while the rest was used for training. Parameters were tuned by ten-fold cross-validation within each training set (see Extended Methods). For the chromatin data, four different classifiers were trained: 1) LASSO logistic regression [29]; 2) Support Vector Machines (SVM) [30] with linear kernel; 3) SVM with radial kernel and 4) Random Forests (RF) [31]. For the sequence data, radial SVMs were not fit. Fig 1C summarizes the modeling strategy. In order to combine the predictions from the separate models, two methods were applied (see Extended Methods for a detailed description): 1) ridge regression (i.e. finding optimal weights for the output from the single classifiers, or “model stacking”) and 2) the weighted sum of the individual output ranks. Predicted values for each one of the input observations are reported in S6 Table, overall performances in S5 Table.

### Genome-wide predictions

For genome-wide predictions, models were fit using 10-fold cross-validation on the entire dataset. After extensive pre-processing of the values of the single features (see Extended Methods), the mouse genome was tiled into gap-less, overlapping 2kb tiles (with a step of 1kb). Tiles overlapping either gene promoters or elements in the training set were discarded. The top 20,000 disjoint elements predicted by each model (or combination) were obtained using an iterative merging strategy (see Extended Methods).

### Assessing variable importance in the LASSO and RF models

For the bootstrap LASSO, 1,000 bootstrap samples of the original data were extracted (see Extended Methods). Model parameters were estimated and selection probabilities for each feature were calculated by dividing the number of non-zero coefficients across bootstrap samples by the total number of bootstrap samples [62]. For the RF, the importance for each variable was defined as the average decrease in accuracy.

### Data and code availability

The Limb-Enhancer Genie (LEG) is an online tool (available at http://leg.lbl.gov/) aimed at facilitating the access to the genome-wide predictions generated in this study. Two separate analysis modes are available. The first one finds the overlap of a set of input regions with the top 10,000 predicted limb-enhancers. This can be used, for example, to scan large regions for potential limb-enhancers. The second one is conceived to assign scores to smaller regions (< = 10kb). For each input region, the highest scoring overlapping 2kb genomic tile is identified and returned along with its score and original coordinates. This also allows scoring of elements overlapping the training data or regions close to promoters, which were excluded from the top 10,000 reported predictions. This second mode of analysis accepts mouse (mm9 and mm10) as well as human (hg19 or hg38) regions. All the predictions along with tracks for the chromatin features and evolutionary conserved TF-binding sites (for the TF-features most correlated with activity in limb) are available on the UCSC Genome Browser [25] (for both mm10 and mm9) via the track hub available at http://portal.nersc.gov/dna/RD/ChIP-Seq/LEG_trackhub/hub.txt. The source code for training and combining the models is available for download at http://github.com/rmonti/limb_enhancer_genie/.

### Data processing in R

All the described data processing steps were performed in the statistical computing environment R v.3.2.1 (www.r-project.org). An overview of the packages used in this study along with references to them is given in the Extended Methods.

### In vivo transgenic reporter assays

Newly tested elements were named according to the nomenclature current in use in the VISTA Enhancer Browser (http://enhancer.lbl.gov/; mm: mouse, hs: human). The elements were amplified from mouse genomic DNA and cloned into an hsp68-*lacZ* expression vector, as previously described [5]. Genomic coordinates are listed in S15 Table. Transgenic mouse assays were conducted as previously described [5,63]. Sample sizes were selected empirically based on past experience of performing transgenic mouse assays for >2,000 total putative enhancers [4]. Mouse embryos were excluded from further analysis if they did not encode the reporter transgene or if the developmental stage was not correct. All transgenic mice were treated with identical experimental conditions. Randomization and experimenter blinding were unnecessary and not performed.

### Experimental model and subject details

Transgenic mouse assays were performed in *Mus musculus* FVB strain mice. The E11.5 developmental stage was considered. Animals of both sexes were used in the analysis. See the previous paragraph for details on sample size selection and randomization strategies.

## Supporting information

S1 Text Extended computational methods. (DOCX)

S1 Fig Overview of the chromatin features used for machine learning across the VISTA elements. The heat map shows the normalized signals. Rows were hierarchically clustered (complete linkage) using one minus Pearson’s Correlation Coefficient as distance; columns were instead clustered based on Euclidean distance. Interesting groups of elements are highlighted by black rectangles and numbered (1–5). Groups 1 and 5, which show over-representation of limb enhancers, are the ones with highest DNase I enrichments. At the same time, groups 2, 3 and 4, which are mainly constituted of elements showing no enhancer activity in the developing limb, show features of either insulators (co-binding of CTCF and the cohesin subunit Smc1a), promoters (high H3K4me3 and H3K9ac) or polycomb-associated heterochromatin (high H3K27me3), respectively.
(TIFF)

S2 Fig CTCF-binding statistics considering the top genome-wide predictions. The top 20,000 genome-wide predictions of models trained on an increasingly larger set of chromatin features (left to right) were binned according to their ranks (best to worst, bins 1 to 10). These bins were overlapped with the CTCF peaks, called at either E10.5 or E11.5. The charts show the fraction of elements in the each bin overlapping CTCF peaks, for different models.
(TIFF)

S3 Fig Receiver-Operating-characteristic and Precision-Recall curves. The ROC (**A**) and the PR (**B**) curves are shown for each one of the indicated models. The average TPR (**A**) or the average precision (**B**) for binned FPR (**A**) or recall (**B**) values across the ten splits are shown. Precision/Recall of the enhancer chromatin states learned by ChromHMM (separately on the two biological replicates) are also shown in (**B**).
(TIFF)

S4 Fig Precision-Recall curves for the combined models (Ridge regression). Curves were calculated over the ten leave-one-out test sets, using the predictions from the combined models (Ridge Regression). (**A-C**) For each one of the indicated combined models (chromatin, sequence, chromatin and sequence) the average precision for binned recall values across the ten splits are shown as a solid line (bin = 0.05). Dashed lines denote the best and the worst performing splits, respectively. (**D**) Overlaid average PR curves from (A-C).
(TIFF)

S5 Fig LEG predictions over the training set in comparison to EnhancerFinder and EMERGE. The distributions of predicted values for the positive (light blue) and negative (light red) regions of the training set are shown for (left to right): EnhancerFinder, EMERGE, combined model (Ridge Regression) and combined model (SOR). For each set of predictions, a boxplot is shown on top of the cumulative densities. Differences were measured using the Kolmogorov–Smirnov statistic (D) and highlighted on top of each boxplot.
(TIFF)

S6 Fig Functional enrichment of the genes in the vicinity of the newly predicted elements. The top 20,000 genome-wide predictions for each model were binned according to their ranks (best to worst, bins 1 to 10). Enrichment for the terms indicated on top of the plots were then calculated using GREAT [42]. The plots show the Fold enrichment (top row) as well as the FDR (bottom row) for all the indicated models across the ten bins.
(TIFF)

S7 Fig Promoter-Capture-C data at the *Hand2* locus. (**A**) UCSC genome browser snapshot of the four topologically associated domains (TADs, top) [64] surrounding the *Hand2* gene locus. CaptureC data using the *Hand2* promoter as viewpoint are shown for both forelimbs and hindlimbs at E11.5 [45]. Significant interactions are shown as black intervals below the raw signals. The four tested regions, along with the top 5,000 predictions from both the Ridge and the Sum Of Ranks (SOR) combined models, and the RefSeq genes in the region are shown. (**B**) UCSC genome browser snapshot of the *Hand2* gene locus, as shown in Fig 5D. The promoter-CaptureC data is shown on top. The probability of being a limb-enhancer (Ridge model) along with the top 5,000 predictions from both the Ridge and the Sum Of Ranks (SOR) combined models are shown. The four elements tested for activity in the developing limbs are highlighted in boxes (green for those showing activity in the limbs at E11.5, red if negative).
(TIFF)

S8 Fig DNase I enrichment significantly improves enhancer prediction performances in tissues other than limb. Box plots showing the AUROC and AUPRC estimated by logistic regression on ten rounds of 5-fold cross validation, considering an increasingly larger set of chromatin features (p300 if available, H3K27ac, DNase I accessibily and DNase I enrichment), in E11.5 midbrain, hindbrain, facial prominence and neural tube. The horizontal lines in the AUPRC plots highlight the value expected by chance, given each specific dataset. ** *p*-value < = 1e-4; *ns* = not significant, p > 0.05 (one-tailed Wilcoxon signed-rank test).
(TIFF)

S9 Fig Effect of sequence conservation on the sequence-based models. (**A**) Bar charts showing the Spearman’s Correlation Coefficient (SCC) of the predicted values (on the training set) *vs* the number of conserved base pairs in each element. (**B**-**C**) Scatterplots for negative and positive examples are shown for one chromatin (SVM radial) and one sequence (RF) models.
(TIFF)

S1 Table Complete list of the VISTA enhancer elements considered in this study. Mm10 coordinates are provided, along with the coordinates and the identifier of each element as indicated at http://enhancer.lbl.gov/ [4]. Whether each element was positive or negative for activity in E11.5 limbs is also indicated. 2,201 out of 2,203 elements were considered throughout the study and for machine learning (ML), so this information is also provided (see Extended Methods).
(XLSX)

S2 Table Sequencing datasets considered in this study (limb). For each dataset, the developmental stage, the tissue and type of experiment (ChIP-seq, DNase-seq, RNA-seq or Whole-Genome-BiSulphite-seq) are indicated. For ChIP-seqs, the target protein is also specified. The data was downloaded either from ENCODE or GEO, so different accessions are provided depending on the source. Every time a control dataset is available, the corresponding accession number(s) are also specified. The Pubmed ID (PMID) of the reference publication is also indicated.
(XLSX)

S3 Table Metadata for the PWMs considered in this study. For each one of the TF-gene expressed in the developing limbs, PWM models were collected from the literature. For each PWM, the table is showing the identifier, the Pubmed ID (PMID) of the reference publication, the gene symbol of the cognate TF and the FPKM for the gene in the developing limbs at E11.5 (as estimated by RNA-seq).
(XLSX)

S4 Table PWMs considered in this study. For each one of the PWM considered in this study (S3 Table), the raw counts are provided. Four records corresponding to the A, C, G and T nucleotides follow the identifier of each PWM (same as in S3 Table, preceded by a “>” sign), with each column representing a position in the motif.
(XLSX)

S5 Table Performances of the different models trained in this study. The mean, median and standard deviation of the AUROC (Area Under the Receiver Operating characteristic Curve) as well as AUPRC (Area Under the Precision Recall Curve) values (as estimated on the ten leave-one-out test sets) are reported for each model.
(XLSX)

S6 Table Predicted values of the models for each VISTA element. For each one of the 2,201 VISTA elements considered, the predicted values are shown. These are in the 0–1 range for all the models except for the SOR, and they were calculated on the same ten leave-one-out test set. The mouse coordinates (mm10), the corresponding VISTA identifier and whether the element is a validated limb-enhancer (positive) or a negative element is indicated.
(XLSX)

S7 Table Predicted TF-binding sites’ clusters. For each one of the VISTA elements considered, the score of the best TF-binding sites’ cluster for each of the cognate TF-gene expressed in the developing limbs (S3 and S4 Tables) is provided.
(XLSX)

S8 Table Statistics for the top predicted *de novo* genome-wide predictions. The total number of tiles (before merging) and the base-pair coverage for the top 10,000 and 20,000 predictions for each one of the models trained in this study are provided.
(XLSX)

S9 Table Summary of chromatin feature importance. The importance of each chromatin feature for enhancer prediction is summarized. Selection probability via Bootstrap LASSO, the coefficients for LASSO and the mean decrease in accuracy for Random Forests are indicated. For the random-forest classifier, mean values were calculated across the ten leave-one-out training/test splits. For LASSO, the mean and median coefficients are calculated across all bootstrap samples.
(XLSX)

S10 Table Summary of sequence feature importance. The importance of each sequence feature for enhancer prediction is summarized, as calculated across the ten leave-one-out training/test splits. Selection probability and coefficients for LASSO, and the mean decrease in accuracy for Random Forests are indicated. Only those features selected by LASSO in at least 1 out of 10 leave-one-out training/test splits are shown.
(XLSX)

S11 Table gkm-SVM performances. The AUROC and AUPRC values for the predictions over the same ten leave-one-out test sets used across this study are provided.
(XLSX)

S12 Table Manually curated list of enhancers active during limb development *in vivo* at stage E11.5 correctly predicted by LEG. The indicated regions were found in the top 1,000 predictions of both combined models but are not part of VISTA. Ranks and score for both the Ridge-regression (RR) and sum-of-ranks (SOR) are provided along with all the relevant information that unambiguously identifies these regions and their corresponding publications. Coordinates are mm10.
(XLSX)

S13 Table Genome-wide predictions (ridge-regression, combined model). Complete list of the top 10,000 *de novo* predictions genome-wide as defined by the combined model based on Ridge-regression (RR). Ranks and scores are provided along with mm10 coordinates.
(XLSX)

S14 Table Genome-wide predictions (sum-of-ranks, combined model). The complete list of the top 10,000 *de novo* predictions genome-wide as defined by the combined model based on the sum-of-ranks (SOR). Ranks and scores are provided along with mm10 coordinates.
(XLSX)

S15 Table Newly predicted elements tested for *in vivo* activity in E11.5 embryos. The VISTA identifier along with the genomic coordinates (both mm9 and mm10), the sequence of the primers, the length of the amplicon and the ranks from both combined models (RR = ridge-regression, SOR = sum-of-ranks) are shown. The table also indicates whether the element was found active in the developing limbs (LacZ staining reproducibility shown in brackets).
(XLSX)

S16 Table H3K27ac-enriched regions in the developing human limbs showing predicted enhancer activity. Regions from four different developmental time points [20] were processed and used as input for LEG (using the “Score short region(s)” analysis type, see Extended Methods). Regions showing an imputed genome-wide rank smaller than 10,000 in either Ridge or SOR model’s predictions are shown. For each region, the imputed rank was inferred from the score of the genome-wide tile (mm10) showing the highest overlap with it. The overlaps were instead calculated using the three indicated sets, considering any kind of overlap (not necessarily the overlap with the tile showing the highest overlap).
(XLSX)

S17 Table Sequencing datasets considered in this study (brain and face). Same as S2 Table.
(XLSX)
