# Supplementary material for: Limb-Enhancer Genie: An accessible resource of accurate enhancer predictions in the developing limb
Source: PLoS Comput Biol. 2017 Aug 21;13(8):e1005720. doi: 10.1371/journal.pcbi.1005720 (PMC5578682; doi:10.1371/journal.pcbi.1005720)
Supplement: S1 Text — (DOCX) [file pcbi.1005720.s001.docx]

**Extended computational methods**

*Validated enhancers from the VISTA Enhancer Browser.* Human and murine validated elements available on January 27, 2016 on the VISTA enhancer browser (<http://enhancer.lbl.gov>) [1] were downloaded and mapped to mm10-coordinates using liftOver [2] (Table S1). LiftOver was run with default parameters except for *minmatch* that was set to 0.1 for mouse to human conversions and to 0.95 for mapping between mm9 and mm10**.** Regions were filtered to be non-overlapping, and those mapping to mm10 but showing long insertions were excluded. This resulted in a curated set of 2,203 elements, among which 2,201 were used for machine learning (two of the VISTA elements showing no enhancer activity were excluded since they showed zero read counts across all chromatin features considered in this study).

*ChIP-seq data analysis.* Limb-specific ChIP-seq datasets spanning three developmental stages (E10.5, E11.5 and E12.5) from the ENCODE project [3] as well as data from four independent limb-centric publications: [4–7] were collected (Table S2). Reads were aligned to the mm10 release of the mouse genome (Dec. 2011, GRCm38) using bowtie2 [8] in *--end-to-end* mode. Only uniquely mapping reads were retained for further analyses. None of the data processing steps in the machine learning framework described below involved peak calling. Peaks were used only in very specific comparative analyses (Figure 1 and Figure S2). MACS v1.4.2 [9] was used. Treatment samples were merged if they constituted technical replicates of the same biological material while control samples were merged indiscriminately for samples coming from the same batch of experiments. For histone modifications (all samples with the exception of CTCF, the cohesin subunit Smc1a and p300) MACS v1.4.2 was run using the *--nomodel* option, otherwise using default settings.

*DNase I Hypersensitivity data analysis.* ENCODE University of Washington DNase I hypersensitivity (*EncodeUwDnase*) raw data was downloaded from the *goldenpath* of the UCSC genome browser [2]. More details about these datasets and their accession numbers are listed in Table S2. The reads coming from samples of fore- and hind- limbs and a set of additional samples from trunk (*headless embryos*) were aligned to mm10 using bowtie2 [8] (refer to the previous paragraph for details). Only uniquely mapping reads were retained for further analysis. As in the case of ChIP-seq datasets, no data processing step in the machine learning framework described below made use of peak-calling. Peaks were called for specific analyses as specified in the text using MACS v1.4.2 [9] using the *--nomodel* option.

*CpG-Methylation data analysis.* CpG-methylation tracks at base-pair resolution were downloaded from the ENCODE Data Collection Center (<http://www.encodeproject.org/>, accession numbers are provided in Table S2) [3]. The bigWig files containing the fraction (0-1) of reads showing methylation at each position throughout the genome were directly parsed during subsequent analyses.

*RNA-seq data analysis.* Limb-specific RNA-seq data from ENCODE were aligned with STAR v2.4.2a [10] to the reference transcriptome (mm10, Ensembl 81 gene-annotation release, [11]) using default settings. Transcripts were quantified with Stringtie v1.0.4 [12] using default settings (Table S2).

*TF-binding motifs.* The position weight matrices (PWMs) collected in [13] (Table S3 and S4) were considered for integration into the sequence-based modeling framework. The gene symbols of the corresponding transcription factors were mapped to the equivalent identifiers in the Ensembl 81 release [11]. In order to reduce the number of features, only those PWMs describing the binding preferences of TFs expressed in E11.5 limbs were included. A TF was considered expressed when showing at least a mean FPKM of 2 as calculated across all RNA-seq replicates (Table S2). This filtering resulted in a set of 291 TFs. The corresponding 823 PWMs were used for the motif detection approach described below.

*Transcription-factor binding sites detection using FIMO.* FIMO (Find Individual Motif Occurrences) is a tool used for detecting potential TF-binding sites, given a PWM and a DNA sequence [14]. After computing the log-likelihood ratio scores for each position in the provided sequence, FIMO converts these scores to *p*-values. Given a certain background model and a score, the *p*-value corresponds to the probability of observing a random sequence of the same length as the motif producing a score at least as good as the one observed. By default, FIMO reports all matches with *p*-value <= 10^-4^. This threshold was kept unchanged. For both the VISTA elements and the genome-wide predictions, sequences were divided into 20 incremental, equally spaced bins according to their GC-content. Mono- and di-nucleotide frequencies were calculated for each one of these bins. FIMO v4.10.2 was then run separately on each bin, using the corresponding nucleotide-frequencies for background estimation.

*Data processing in R.* All the described data-processing steps were performed using the statistical computing environment R v.3.2.1 ([www.r-project.org](http://www.r-project.org)). An overview of the packages used, along with a reference and the information about the context in which they were used is given in the table below.

| **Package** | **Version** | **Use** | **Reference** |
| --- | --- | --- | --- |
| biomaRt | 2.24.1 | Interface to BioMart Databases | [15] |
| BSgenome | 1.36.3 | Full Genome Sequences | Bioconductor – Pagès (2016) |
| caret | 6.0-64 | Parameter Tuning, Data Partitioning, Logistic Regression | [http://www.jstatsoft.org/article/view/v028i05](https://www.jstatsoft.org/article/view/v028i05) |
| doMC | 1.3.4 | Parallel Computing | CRAN – Weston (2015) |
| data.table | 1.9.4 | Data Handling | CRAN - Dowle et al. (2014) |
| e1071 | 1.6-7 | SVM Classifiers | CRAN - Meyer et al. (2015) |
| GenomicAlignments | 1.4.1 | Read Counting | [16] |
| GenomicRanges | 1.20.5 | Genomic Intervals Manipulation | [16] |
| ggplot2 | 2.0.0 | Plotting | CRAN - Wickham et al. (2009) |
| glmnet | 2.0-2 | LASSO-, Ridge-regularized models | CRAN - Friedman et al. (2010) |
| gplots | 2.17.0 | Heatmaps | CRAN - Warnes et al. (2015) |
| PRROC | 1.1 | AUROC, AUPRC Calculation | [17] |
| randomForest | 4.6-10 | Random Forest | CRAN - Liaw and Wiener (2002) |
| RColorBrewer | 1.1-2 | ColorBrewer Palettes | CRAN - Neuwirth (2014) |
| rtracklayer | 1.28.9 | LiftOver, Data Import/Export | [18] |

*Calculation of enrichment of chromatin features over genomic regions.* The 2,201 mm10-mapped VISTA elements were expanded to a minimum length of 2kb around their center (the expansion affected 732 elements, 33%). The number of reads mapping to each element from ChIP-seq, and DNase I samples were counted, resulting in a count-matrix with regions on the rows and assays on the columns. A pseudocount was added to all values before normalization to reads per kilobase per million mapped reads (RPKM), which takes into consideration the element width and the number of uniquely mapped reads per assay.

Considering ChIP-seq samples, the enrichments over the corresponding control samples (input DNA) were calculated. In order to reduce the noise in the estimation of the coverage in the input DNA over elements as small as 2kb, a larger window (+/- 5 kb from the center of the element) were considered for background estimation. For DNase I, fold enrichments of the hind- and forelimb signals over the *headless-embryo* signals were included as additional features. Resulting values were log2-transformed. Considering CpG-methylation, the average base-pair resolution fraction inside each region was determined. In cases where there were no CpG di-nucleotides and therefore no data, the value was imputed as the average of all remaining regions. Replicates coming from the same studies were averaged before conversion to *z*-scores. If a replicate within a study had a signal to noise ratio (estimated by the number of reads under the called peaks relative to the total number of mapped reads in the sample) lower than half that of the best replicate within that study, it was excluded. Effectively, this filtering step only led to the exclusion of one replicate (GSM1036122, CTCF) from the analysis.

A final set of 31 distinct “chromatin-features” from functional genomics studies were incorporated into the machine-learning framework described later in the text.

*Estimation of TF-binding sites’ clusters for limb-expressed TFs from DNA sequence.* PWMs were limited to those representing binding preferences of TFs potentially expressed in the developing limb (see paragraph above). Putative TF-binding sites were identified using FIMO as described above. Either the mouse or the human sequence was scanned according to which version was tested *in vivo* (Table S1, *mm* or *hs* VISTA identifiers). For some of the TFs no binding sites exceeded the defined *p*-value threshold, leading to their exclusion from the analysis. For the remaining 289 TFs, features were calculated as follows: when multiple predicted binding sites for the same TF overlapped (either from the same PWM or from different PWM), only the highest scoring site (the one with the lowest *p*-value) was retained. For each one of the genomic locations overlapping a binding site, the *phastcons* [19] scores at base-pair resolution were parsed (60way placental for mm10, 46way placental for hg19) and averaged. Each site was assigned a score according to the following formula: average phastcons * -log10(FIMO *p*-value). This takes into account both the theoretical affinity for the TF to the site (based on the published binding models) and the evolutionary conservation. The highest-scoring 500bp cluster of binding sites for each TF was identified within each element considered (i.e. tiles over the genome, or VISTA enhancers). This was determined by first identifying all the possible 500bp clusters, then picking the cluster showing the highest sum of scores for the binding sites for the TF considered. The devised score is proportional to the number of evolutionary conserved, putative TFBSs inside the highest-scoring cluster in the region. Features were finally normalized to *z*-scores.

Besides considering the TF-binding sites’ clusters as input features for the model, the overall CG-content of each region was also measured and taken into account.

*Training the models and performance assessment.* VISTA elements showing activity in the limb were defined as the positive class while all the other elements (those showing activity in any other tissue but not in limb or showing no activity at all) constituted the negative class.

Ten equally sized splits were generated for the 2,201 VISTA elements using a stratified sampling strategy, i.e. maintaining the ratio of positive to negative observations of the entire set. Nine out of ten splits were used as training set, while the remaining one was used as independent test set to assess model performance. This was repeated ten times for all possible combinations of nine training vs one test set. 10-fold cross-validation was used for parameter tuning within each training set. For the chromatin data, four different classifiers were trained: 1) a LASSO logistic regression classifier [20]; 2) a Support Vector Machine (SVM) [21] with linear kernel; 3) an SVM with radial kernel and 4) a Random Forest classifier [22]. For the sequence data, radial SVMs were not fit, resulting in seven different models in total (four for chromatin and three for sequence).

The *lambda* parameter for the LASSO, the cost-parameter *C* for the linear and radial SVM, and the *gamma* parameter for the radial SVM were tuned by maximizing the AUROC during 10-fold cross-validation on each training set. For the SVMs and the LASSO, the total class-weights were adjusted to account for the imbalance in the representation of the two classes (a weight corresponding to N_negative_/N_positive_ – where N is the total number of elements in the class in the training set – was assigned to each element in the positive class). For the random forest, stratified sampling in a 1 to 1 ratio (positive vs negative) was enforced, and the number of trees was set to 1,000. In order to perform an independent evaluation of the performances of each model and to be able to compare the results across different models, the optimized models were finally used to predict the corresponding test sets.

In order to combine the predictions of the separate models, two methods were used: 1) ridge regression (i.e. finding optimal weights for the output from each classifier, ‘model stacking’, see below) and 2) a weighted sum of ranks-approach. Ten ridge regression models were fit on the predicted values (computed over combinations of the test sets) of the seven models. The class weights were again adjusted to be equal and the optimal *lambda* shrinkage-parameter was found by minimizing the mean squared error using 10-fold cross-validation and choosing the largest value of *Lambda* such that the error was within one standard error of the minimum. In order to compute the weighted sum of ranks, the predicted values from each model were converted into ranks and a final score for each element was calculated using the following formula: mean(ranks across chromatin models)+0.3*mean(ranks across sequence models). Given the prior knowledge about the poorer performances of the sequence-based models, an arbitrary weight of 0.3 was assigned to the average rank of the sequence-based predictions without any further optimization.

For the specific R packages used, refer to the paragraph and table above. The code to perform these operations (training and combining the models) is available for download at [http://github.com/rmonti/limb_enhancer_genie](https://github.com/rmonti/limb_enhancer_genie)/.

Genome-wide predictions. For genome-wide predictions, models were fit using 10-fold cross-validation on the entire dataset. Parameters were optimized as described in the previous section. The mouse genome was tiled into gap-less overlapping 2kbp tiles (with a step of 1kbp).

Data was collected and transformed as described in the previous paragraphs. The tiles showing no reads across all the considered chromatin datasets were discarded. Even though 10kbp windows were used for background estimation, in order to decrease the influence of low, noisy measurements when calculating fold enrichments in regions of poor mappability, the signal from each one of the control samples (input) was adjusted as follows:

- the sample-specific median and standard deviation of the genome-wide tiles was calculated;
- those windows showing a signal lower than the median minus 1.5 standard deviations were identified and their value was replaced with the sample-specific median minus 1.5 standard deviations.

This transformation affected on average 4.4% of the genome-wide tiles for each control-sample. Following a similar logic, when calculating enrichments of hindlimb- or forelimb-DNase I over headless-embryo DNase I, those tiles showing values below the first decile of all the tiles in the headless-embryo samples were replaced by the value of the first decile.

Additionally, those tiles close to known gene promoters ([-5000bp, +2000bp] around TSSs of Ensembl genes release 82 [11]) as well as those ones overlapping the original 2,201 elements from VISTA used to train the models were discarded.

*Ranking genome-wide predictions.* Genome-wide predictions were ranked according to the single model outputs (decision values for the SVMs, probabilities for the LASSO and the ridge regression, fraction of votes for the random forests, or the weighted sum of ranks itself). The top 20,000 distinct elements were extracted separately for each model or combination of models. In order to define these highest-ranking elements, the following procedure was applied:

1. identification of the highest scoring 20,000 tiles;
2. merge overlapping tiles;
3. assign each region the score of the highest ranking tile overlapping it;
4. re-iterate 1-3 but add more of the highest scoring tiles incrementally, until 20,000 distinct, non-overlapping regions were obtained.

The total number of tiles considered as well as the final coverage for both the top 10,000 and 20,000 regions are reported in Table S8.

*Assessing variable importance in the LASSO and RF models.* *Bootstrap LASSO* – One thousand bootstrap samples of the original data were extracted. As described in the previous paragraph, class weights were adjusted to account for the imbalance between positive and negative samples. The optimal shrinkage parameter *lambda* was estimated using 10-fold cross-validation, as described above for the Ridge-model. Model parameters at these values of *lambda* were extracted and selection probabilities were calculated by dividing the number of non-zero coefficients for each feature across bootstrap samples by the total number of bootstrap samples [23].

*Random Forest* – The importance for each variable was evaluated as the mean decrease in accuracy (the more important a variable is, the biggest the drop in the out-of-bag accuracy of the classifier when the values for that variable are randomly shuffled).

*Functional enrichment analysis of genome-wide predicted limb enhancers.* Considering each model separately, the top 20,000 distinct regions were binned into ten sequential groups according to their rank (the first one containing the 2,000 highest ranking regions, the second one those element form rank 2,001 to 4,000, and so on and so forth). These datasets were submitted to GREAT [24] and the enrichments along with the corresponding statistical significance for ontological terms of interest retained.

*Pubmed querying.* The total number of publications related to a given gene was estimated by the sum of the results returned by Pubmed for the query “*gene symbol*” (where *gene symbol* is the official symbol for the given gene in mouse). Only those genes showing 25% or more of their total publications associated to keyword “development” were retained. Of those, the overall number of citations retrieved by combining the keywords “*gene symbol*”, “development” and “limb” was calculated. Pubmed queries were run systematically via R, taking advantage of the packages *RCurl* and *XML* (Temple Lang 2016).

*gkm-SVM predictions*. The *gkmSVM-R* package [25,26] was used to train the models. Parameters of the R function *gkmsvm_trainCV* were set to default, except for *C* that was tuned using values 1, 2, 4, 8 and 16.

*ChromHMM-derived enhancers predictions*. ChIP-seqs for eight histone modifications (H3K4me1, H3K4me2, H3K4me3, H3K27ac, H3K9ac, H3K36me3, H3K27me3, H3K9me3) for E11.5 limbs were considered. Aligned reads for these datasets and E11.5 input DNA were downloaded from [http://www.encodeproject.org/](https://www.encodeproject.org/). Signal binarization was performed using the *binarizeBam* function of ChromHMM [27], with default parameters. Two different 15-states models were learned separately from the available biological replicates using the *LearnModel* function, with default parameters [28]. Two strategies (as described in [28]) were applied to determine the optimal number of states describing the data; both of them converged on 15. Two of these 15 states (states 5 and 6 in [28]) were found to enrich for known features of strong enhancers (high levels of H3K27ac and H3K4me1/me2). The regions classified in either of these two states were used as predicted limb enhancers. Each one of the considered VISTA elements (Table S1) was annotated as overlapping any of these predictions if the element was covered by a ChromHMM-prediction for at least 50% of its length.

*Promoter-Capture-C data processing*. Data for the viewpoint close to the annotated *Hand2* gene promoter [29] from both forelimbs and hindlimbs of E11.5 embryos were downloaded from the GEO ([GSE84792](https://www.ncbi.nlm.nih.gov/gds/?term=GSE84792%5BAccession%5D)). The normalized signals (smoothed with a 5 kbp window) and the coordinates of the interacting peaks (0.95 threshold) were lifted to mm10 from mm9 using liftOver (*minmatch*=0.95).

*Scanning the H3K27ac-enriched regions in the developing human limbs for predicted limb enhancers.* The H3K27ac-enriched regions at multiple developmental stages (E33, E41, E44 and E47) [4] were downloaded from the GEO ([GSE42413](https://www.ncbi.nlm.nih.gov/gds/?term=GSE42413%5BAccession%5D)). ChIP-seq peaks from all four developmental stages were intersected. Only regions consistently found across all four were kept for further analyses. After that, regions shorter than 2kb were expanded to 2kb and merged if overlapping. Finally, regions longer than 10kb were split into smaller intervals of ~2kb. The resulting list was upload to <http://leg.lbl.gov/> and submitted to the “Score Short Region(s)” analysis mode. Results from both scoring methods (Ridge and SOR) were saved. Elements that did not map to the mm10 mouse genome (using liftOver) were excluded. Those showing instead an imputed genome-wide rank smaller than 10,000 in either the Ridge or SOR model’s predictions were retained. This entire workflow is part of one of the tutorials available at <http://leg.lbl.gov/> (Vignette #2).

*Models trained on midbrain, hindbrain, facial-prominence and neural-tube VISTA enhancers*. Raw data analysis for the p300 datasets was performed as described earlier in the text. For H3K27ac ChIP-seq and Dnase I hypersensitivity assays from ENCODE (see Table S17) the aligned reads were downloaded from [http://www.encodeproject.org](https://www.encodeproject.org)/. For each tissue, we defined the training sets based on VISTA annotations [1] as described earlier for limb. Features were also calculated as described in earlier paragraphs.

Logistic regression models of increasing complexity were fit and the performance assessed through ten rounds of 5-fold cross-validation for each tissue. The same ten cross-validation splits were consistently employed for the different feature sets.

*Data Availability at the UCSC Genome Browser.* Prediction tracks tiling the entire genome are available in the UCSC genome browser for both the mm9 and the mm10 build of the mouse genome. The hub for mm10 additionally allows the complete visualization of the ChIP-seq, DNase I hypersensitity and CpG-methylation tracks that were used for feature calculation along with the tracks highlighting the peaks called by MACS. Highly conserved TFBS (average phastcons >= 0.5) of TFs whose features showed at least 0.05 Pearson’s Correlation with the class-labels (encoded as 0/1) were included as an additional track to aid data-exploration.

*LEG website*. The Limb Enhancer Genie is an online tool developed to facilitate data-access to the predictions from either the combined ridge-model or the weighted sum of ranks generated in this study.

Two separate analysis modes are available that serve slightly different purposes. The first one simply finds overlaps of a single or a set of input regions with the top 10,000 predicted limb-enhancers. This can be used to scan large regions, e.g. TADs or introns, for potential limb-enhancers. The second one is meant to assign scores to smaller regions (<=10kb). For each input region it finds the highest scoring overlapping 2kb tile (used for genome-wide predictions, see previous paragraphs) and returns the scores along with the coordinates of the windows. The minimum overlap required is 1kb and input query regions shorter than 1kb are expanded to 1kb prior to performing the overlap. This mode of analysis can be used if a score for every supplied region is desired. If run in this mode, LEG also returns the scores for those elements overlapping the training data, or regions close to promoters. In order to find cross-species conserved enhancers, this second mode of analysis accepts mouse (mm9 and mm10) as well as human (hg19 or hg38) coordinates via liftOver mapping [2] (*minmatch* set to 0.1 for mouse to human conversion). The application was implemented as an R-Shiny app (v0.13.2) using the *rtracklayer* (v1.30.4), *GenomicRanges* (v1.22.4) and *data.table* (v1.9.6) packages.

*Code availability.* The source code for training and combining the models is available for download at [http://github.com/rmonti/limb_enhancer_genie/](https://github.com/rmonti/limb_enhancer_genie/).

*References*

1. Visel A, Minovitsky S, Dubchak I, Pennacchio LA. VISTA Enhancer Browser--a database of tissue-specific human enhancers. Nucleic Acids Res. 2007;35: D88–92. doi:10.1093/nar/gkl822

2. Speir ML, Zweig AS, Rosenbloom KR, Raney BJ, Paten B, Nejad P, et al. The UCSC Genome Browser database: 2016 update. Nucleic Acids Res. Oxford University Press; 2016;44: D717–D725. doi:10.1093/nar/gkv1275

3. Bernstein BE, Birney E, Dunham I, Green ED, Gunter C, Snyder M. An integrated encyclopedia of DNA elements in the human genome. Nature. Nature Publishing Group, a division of Macmillan Publishers Limited. All Rights Reserved.; 2012;489: 57–74. doi:10.1038/nature11247

4. Cotney J, Leng J, Yin J, Reilly SK, DeMare LE, Emera D, et al. The evolution of lineage-specific regulatory activities in the human embryonic limb. Cell. 2013;154: 185–96. doi:10.1016/j.cell.2013.05.056

5. Cotney J, Leng J, Oh S, DeMare LE, Reilly SK, Gerstein MB, et al. Chromatin state signatures associated with tissue-specific gene expression and enhancer activity in the embryonic limb. Genome Res. 2012;22: 1069–80. doi:10.1101/gr.129817.111

6. DeMare LE, Leng J, Cotney J, Reilly SK, Yin J, Sarro R, et al. The genomic landscape of cohesin-associated chromatin interactions. Genome Res. 2013;23: 1224–34. doi:10.1101/gr.156570.113

7. VanderMeer JE, Smith RP, Jones SL, Ahituv N. Genome-wide identification of signaling center enhancers in the developing limb. Development. 2014;141: 4194–8. doi:10.1242/dev.110965

8. Langmead B, Salzberg SL. Fast gapped-read alignment with Bowtie 2. Nat Methods. 2012;9: 357–9. doi:10.1038/nmeth.1923

9. Zhang Y, Liu T, Meyer CA, Eeckhoute J, Johnson DS, Bernstein BE, et al. Model-based analysis of ChIP-Seq (MACS). Genome Biol. 2008;9: R137. doi:10.1186/gb-2008-9-9-r137

10. Dobin A, Davis CA, Schlesinger F, Drenkow J, Zaleski C, Jha S, et al. STAR: ultrafast universal RNA-seq aligner. Bioinformatics. 2013;29: 15–21. doi:10.1093/bioinformatics/bts635

11. Cunningham F, Amode MR, Barrell D, Beal K, Billis K, Brent S, et al. Ensembl 2015. Nucleic Acids Res. 2015;43: D662–9. doi:10.1093/nar/gku1010

12. Pertea M, Pertea GM, Antonescu CM, Chang T-C, Mendell JT, Salzberg SL. StringTie enables improved reconstruction of a transcriptome from RNA-seq reads. Nat Biotechnol. Nature Research; 2015;33: 290–295. doi:10.1038/nbt.3122

13. Barozzi I, Simonatto M, Bonifacio S, Yang L, Rohs R, Ghisletti S, et al. Coregulation of Transcription Factor Binding and Nucleosome Occupancy through DNA Features of Mammalian Enhancers. Mol Cell. Cell Press; 2014;54: 844–857.

14. Grant CE, Bailey TL, Noble WS. FIMO: scanning for occurrences of a given motif. Bioinformatics. 2011;27: 1017–8. doi:10.1093/bioinformatics/btr064

15. Durinck S, Spellman PT, Birney E, Huber W. Mapping identifiers for the integration of genomic datasets with the R/Bioconductor package biomaRt. Nat Protoc. NIH Public Access; 2009;4: 1184–91. doi:10.1038/nprot.2009.97

16. Lawrence M, Huber W, Pagès H, Aboyoun P, Carlson M, Gentleman R, et al. Software for Computing and Annotating Genomic Ranges. Prlic A, editor. PLoS Comput Biol. Public Library of Science; 2013;9: e1003118. doi:10.1371/journal.pcbi.1003118

17. Grau J, Grosse I, Keilwagen J. PRROC: computing and visualizing precision-recall and receiver operating characteristic curves in R. Bioinformatics. Oxford University Press; 2015;31: 2595–7. doi:10.1093/bioinformatics/btv153

18. Lawrence M, Gentleman R, Carey V. rtracklayer: an R package for interfacing with genome browsers. Bioinformatics. Oxford University Press; 2009;25: 1841–2. doi:10.1093/bioinformatics/btp328

19. Siepel A, Bejerano G, Pedersen JS, Hinrichs AS, Hou M, Rosenbloom K, et al. Evolutionarily conserved elements in vertebrate, insect, worm, and yeast genomes. Genome Res. Cold Spring Harbor Laboratory Press; 2005;15: 1034–1050. doi:10.1101/GR.3715005

20. Tibshirani R. Regression Shrinkage and Selection via the Lasso. J R Stat Soc Ser B Stat Methodol. 2007;58: 267–288. doi:10.1111/j.1467-9868.2011.00771.x

21. Cortes C, Vapnik V. Support Vector Networks. Mach Learn. Kluwer Academic Publishers; 1995;20: 273~–~297. doi:10.1007/BF00994018

22. Breiman L. Random forests. Mach Learn. Kluwer Academic Publishers; 2001;45: 5–32. doi:10.1023/A:1010933404324

23. Comoglio F, Paro R. Combinatorial modeling of chromatin features quantitatively predicts DNA replication timing in Drosophila. Michor F, editor. PLoS Comput Biol. Public Library of Science; 2014;10: e1003419. doi:10.1371/journal.pcbi.1003419

24. McLean CY, Bristor D, Hiller M, Clarke SL, Schaar BT, Lowe CB, et al. GREAT improves functional interpretation of cis-regulatory regions. Nat Biotechnol. Nature Publishing Group; 2010;28: 495–501. doi:10.1038/nbt.1630

25. Ghandi M, Lee D, Mohammad-Noori M, Beer MA. Enhanced regulatory sequence prediction using gapped k-mer features. PLoS Comput Biol. 2014;10: e1003711. doi:10.1371/journal.pcbi.1003711

26. Ghandi M, Mohammad-Noori M, Ghareghani N, Lee D, Garraway L, Beer MA. gkmSVM: an R package for gapped-kmer SVM. Bioinformatics. 2016;32: 2205–2207. doi:10.1093/bioinformatics/btw203

27. Ernst J, Kellis M. Discovery and characterization of chromatin states for systematic annotation of the human genome. Nat Biotechnol. Nature Research; 2010;28: 817–825. doi:10.1038/nbt.1662

28. Gorkin D, Barozzi I, Zhang Y, Lee AY, Lee B, Zhao Y, et al. Systematic mapping of chromatin state landscapes during mouse development. bioRxiv. 2017; Available: http://www.biorxiv.org/content/early/2017/07/21/166652

29. Andrey G, Schöpflin R, Jerković I, Heinrich V, Ibrahim DM, Paliou C, et al. Characterization of hundreds of regulatory landscapes in developing limbs reveals two regimes of chromatin folding. Genome Res. Cold Spring Harbor Laboratory Press; 2017;27: 223–233. doi:10.1101/gr.213066.116
