# Supplementary figures and images for: Limb-Enhancer Genie: An accessible resource of accurate enhancer predictions in the developing limb
Source: PLoS Comput Biol. 2017 Aug 21;13(8):e1005720. doi: 10.1371/journal.pcbi.1005720 (PMC5578682; doi:10.1371/journal.pcbi.1005720)

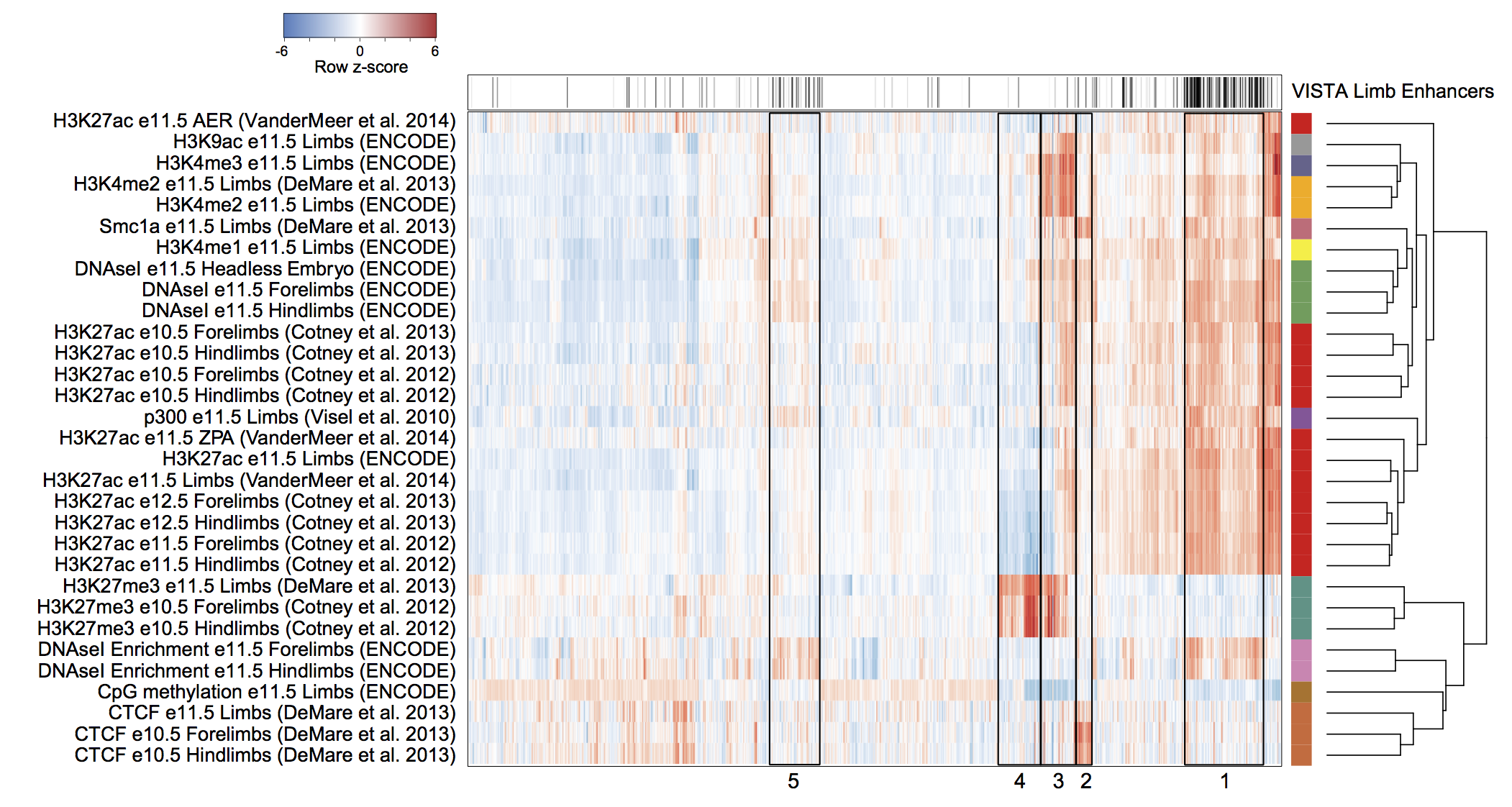

Supplement: S1 Fig — The heat map shows the normalized signals. Rows were hierarchically clustered (complete linkage) using one minus Pearson’s Correlation Coefficient as distance; columns were instead clustered based on Euclidean distance. Interesting groups of elements are highlighted by black rectangles and numbered (1–5). Groups 1 and 5, which show over-representation of limb enhancers, are the ones with highest DNase I enrichments. At the same time, groups 2, 3 and 4, which are mainly constituted of elements showing no enhancer activity in the developing limb, show features of either insulators (co-binding of CTCF and the cohesin subunit Smc1a), promoters (high H3K4me3 and H3K9ac) or polycomb-associated heterochromatin (high H3K27me3), respectively. (TIFF) [file pcbi.1005720.s002.tiff]

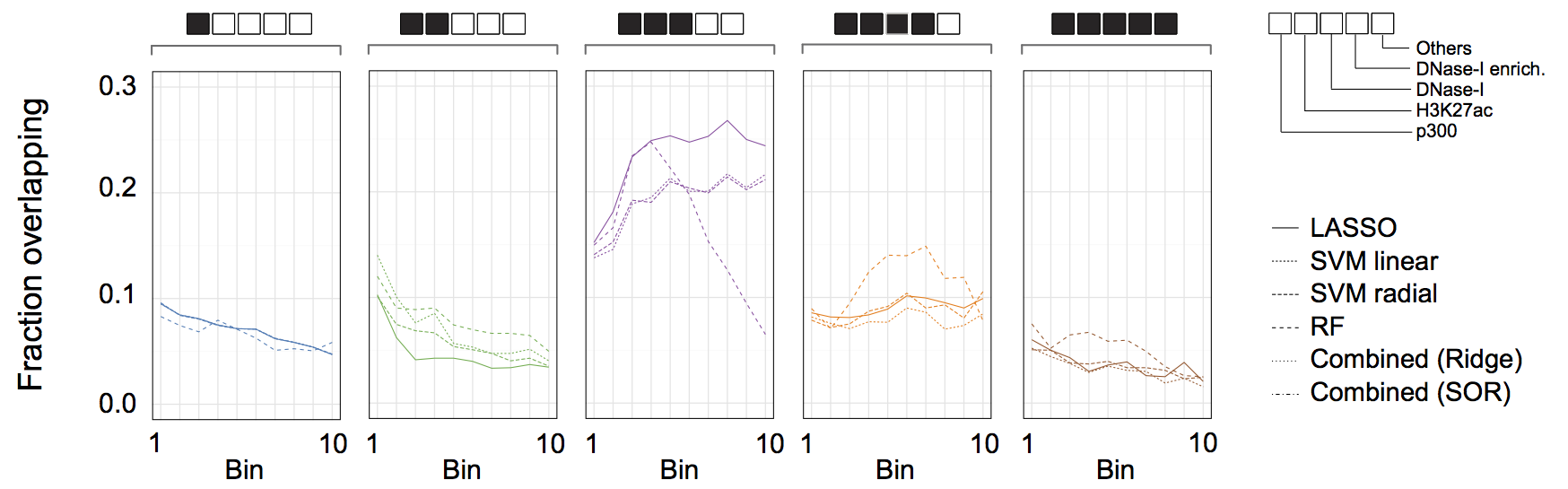

Supplement: S2 Fig — The top 20,000 genome-wide predictions of models trained on an increasingly larger set of chromatin features (left to right) were binned according to their ranks (best to worst, bins 1 to 10). These bins were overlapped with the CTCF peaks, called at either E10.5 or E11.5. The charts show the fraction of elements in the each bin overlapping CTCF peaks, for different models. (TIFF) [file pcbi.1005720.s003.tiff]

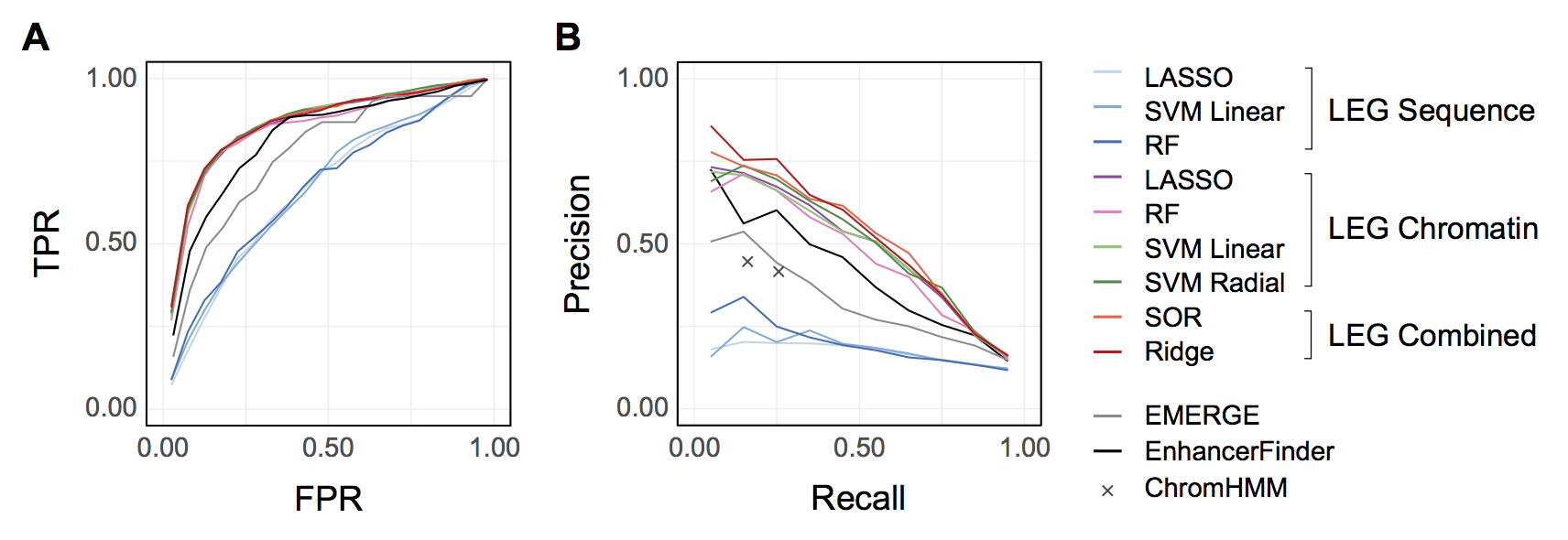

Supplement: S3 Fig — The ROC (A) and the PR (B) curves are shown for each one of the indicated models. The average TPR (A) or the average precision (B) for binned FPR (A) or recall (B) values across the ten splits are shown. Precision/Recall of the enhancer chromatin states learned by ChromHMM (separately on the two biological replicates) are also shown in (B). (TIFF) [file pcbi.1005720.s004.tiff]

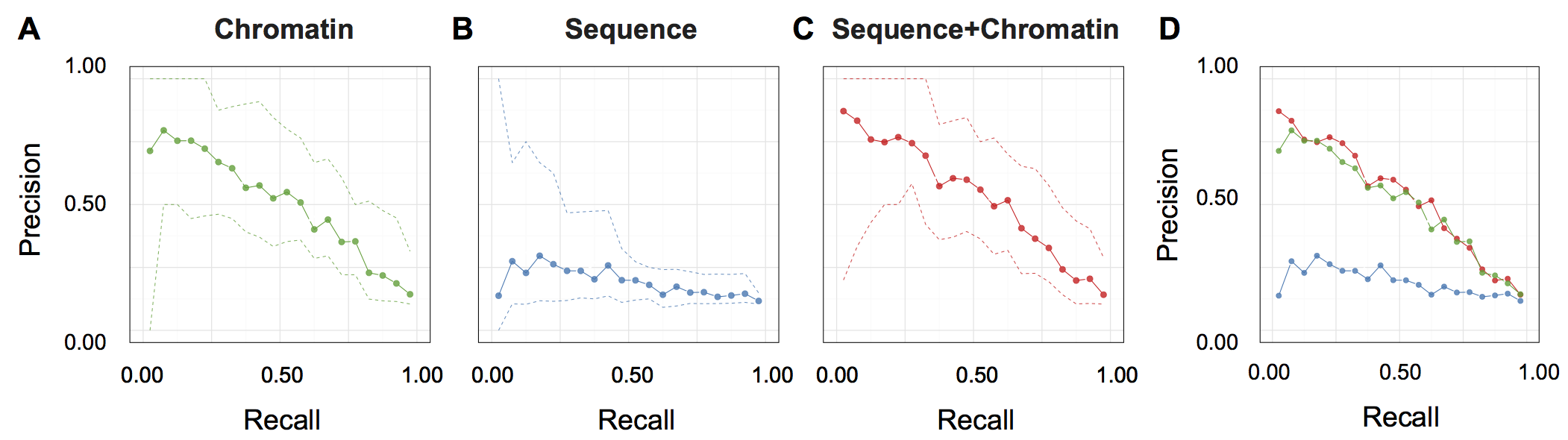

Supplement: S4 Fig — Curves were calculated over the ten leave-one-out test sets, using the predictions from the combined models (Ridge Regression). (A-C) For each one of the indicated combined models (chromatin, sequence, chromatin and sequence) the average precision for binned recall values across the ten splits are shown as a solid line (bin = 0.05). Dashed lines denote the best and the worst performing splits, respectively. (D) Overlaid average PR curves from (A-C). (TIFF) [file pcbi.1005720.s005.tiff]

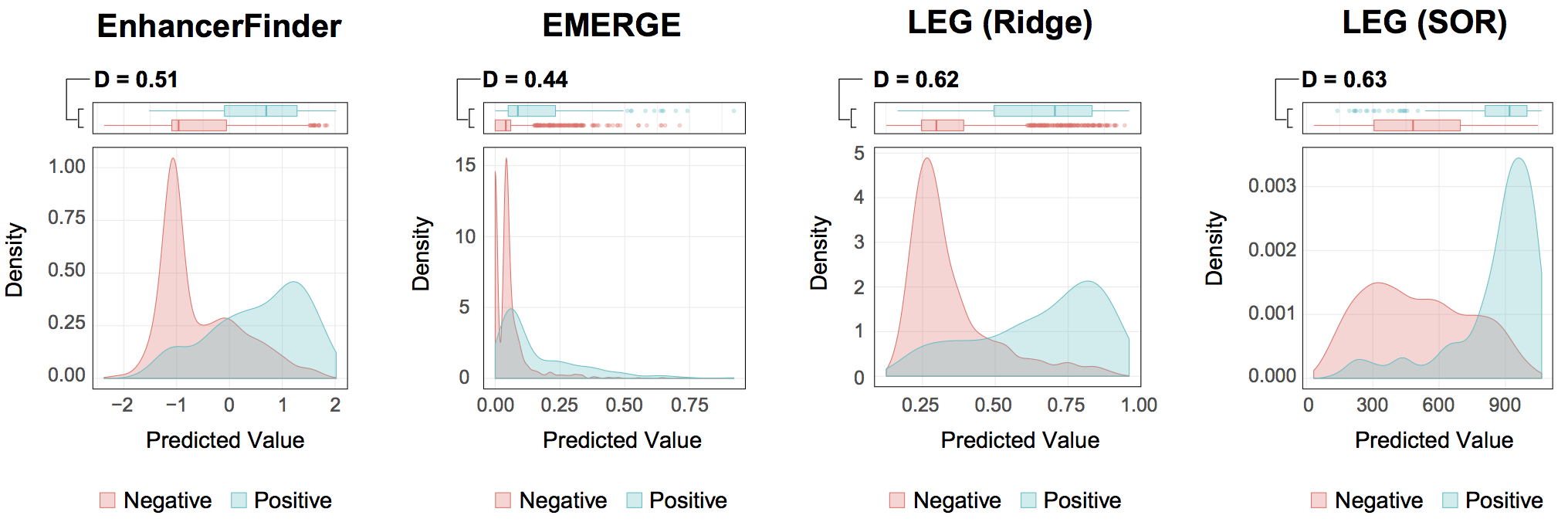

Supplement: S5 Fig — The distributions of predicted values for the positive (light blue) and negative (light red) regions of the training set are shown for (left to right): EnhancerFinder, EMERGE, combined model (Ridge Regression) and combined model (SOR). For each set of predictions, a boxplot is shown on top of the cumulative densities. Differences were measured using the Kolmogorov–Smirnov statistic (D) and highlighted on top of each boxplot. (TIFF) [file pcbi.1005720.s006.tiff]

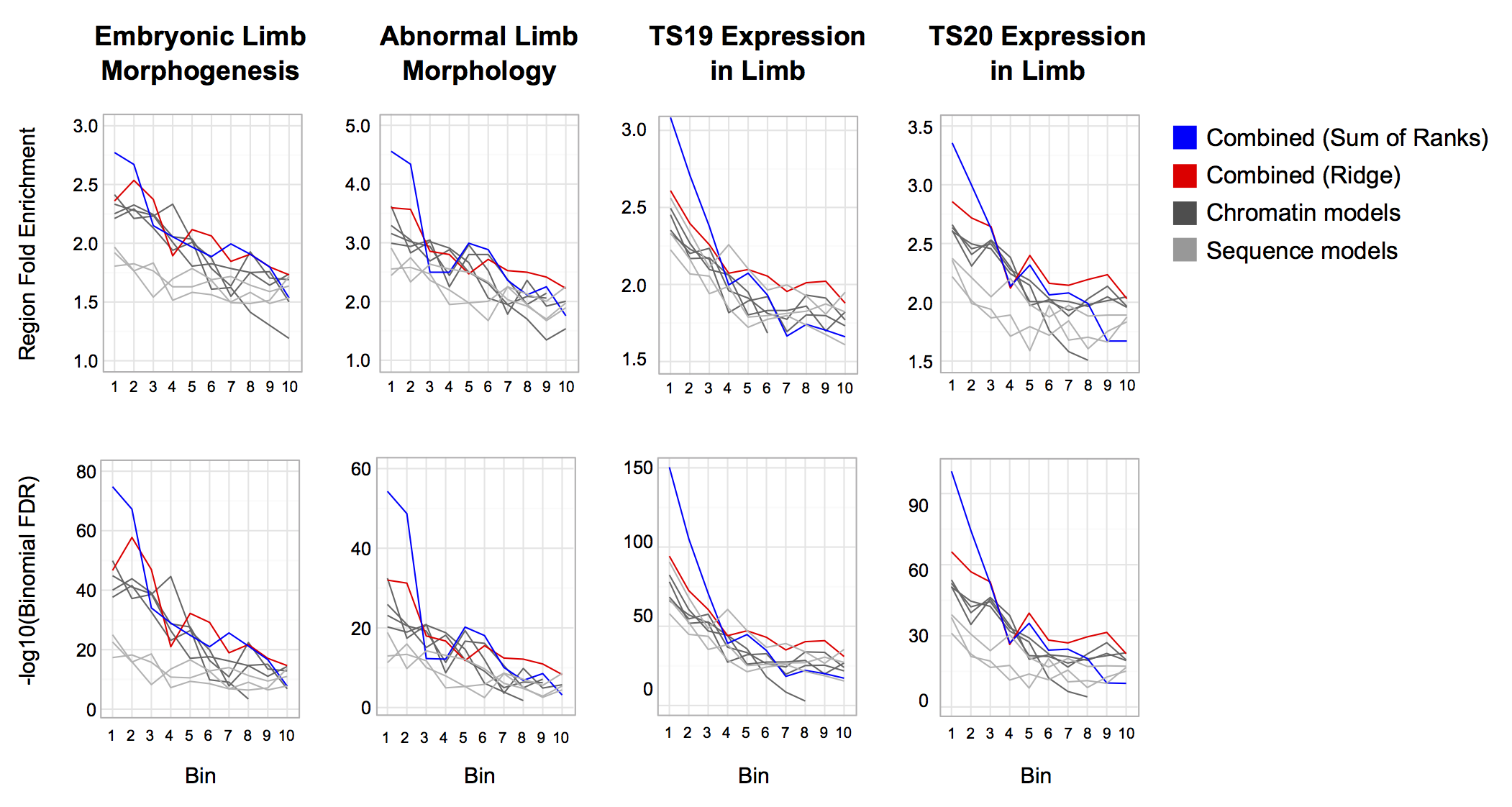

Supplement: S6 Fig — The top 20,000 genome-wide predictions for each model were binned according to their ranks (best to worst, bins 1 to 10). Enrichment for the terms indicated on top of the plots were then calculated using GREAT [42]. The plots show the Fold enrichment (top row) as well as the FDR (bottom row) for all the indicated models across the ten bins. (TIFF) [file pcbi.1005720.s007.tiff]

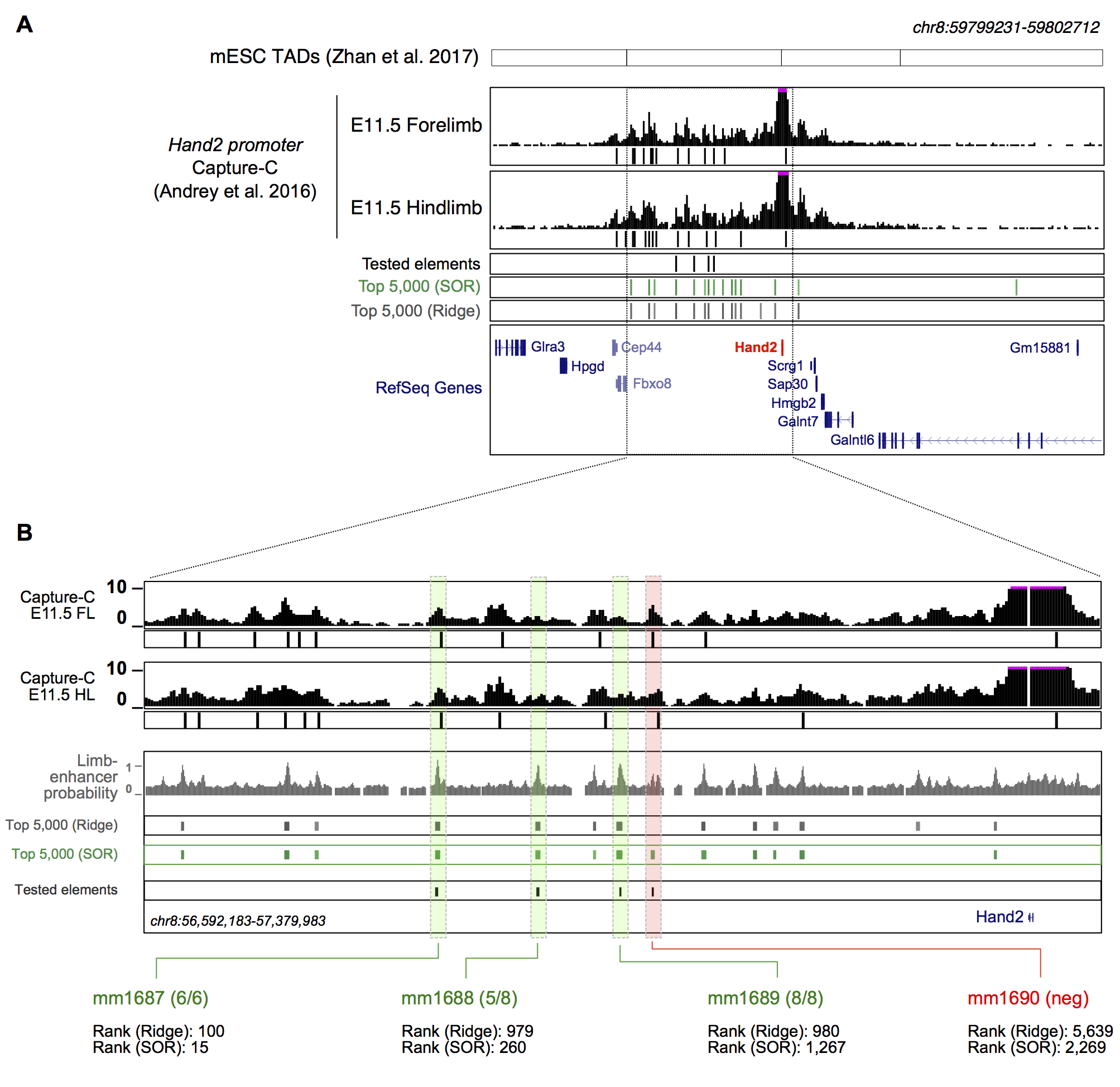

Supplement: S7 Fig — (A) UCSC genome browser snapshot of the four topologically associated domains (TADs, top) [64] surrounding the Hand2 gene locus. CaptureC data using the Hand2 promoter as viewpoint are shown for both forelimbs and hindlimbs at E11.5 [45]. Significant interactions are shown as black intervals below the raw signals. The four tested regions, along with the top 5,000 predictions from both the Ridge and the Sum Of Ranks (SOR) combined models, and the RefSeq genes in the region are shown. (B) UCSC genome browser snapshot of the Hand2 gene locus, as shown in Fig 5D. The promoter-CaptureC data is shown on top. The probability of being a limb-enhancer (Ridge model) along with the top 5,000 predictions from both the Ridge and the Sum Of Ranks (SOR) combined models are shown. The four elements tested for activity in the developing limbs are highlighted in boxes (green for those showing activity in the limbs at E11.5, red if negative). (TIFF) [file pcbi.1005720.s008.tiff]

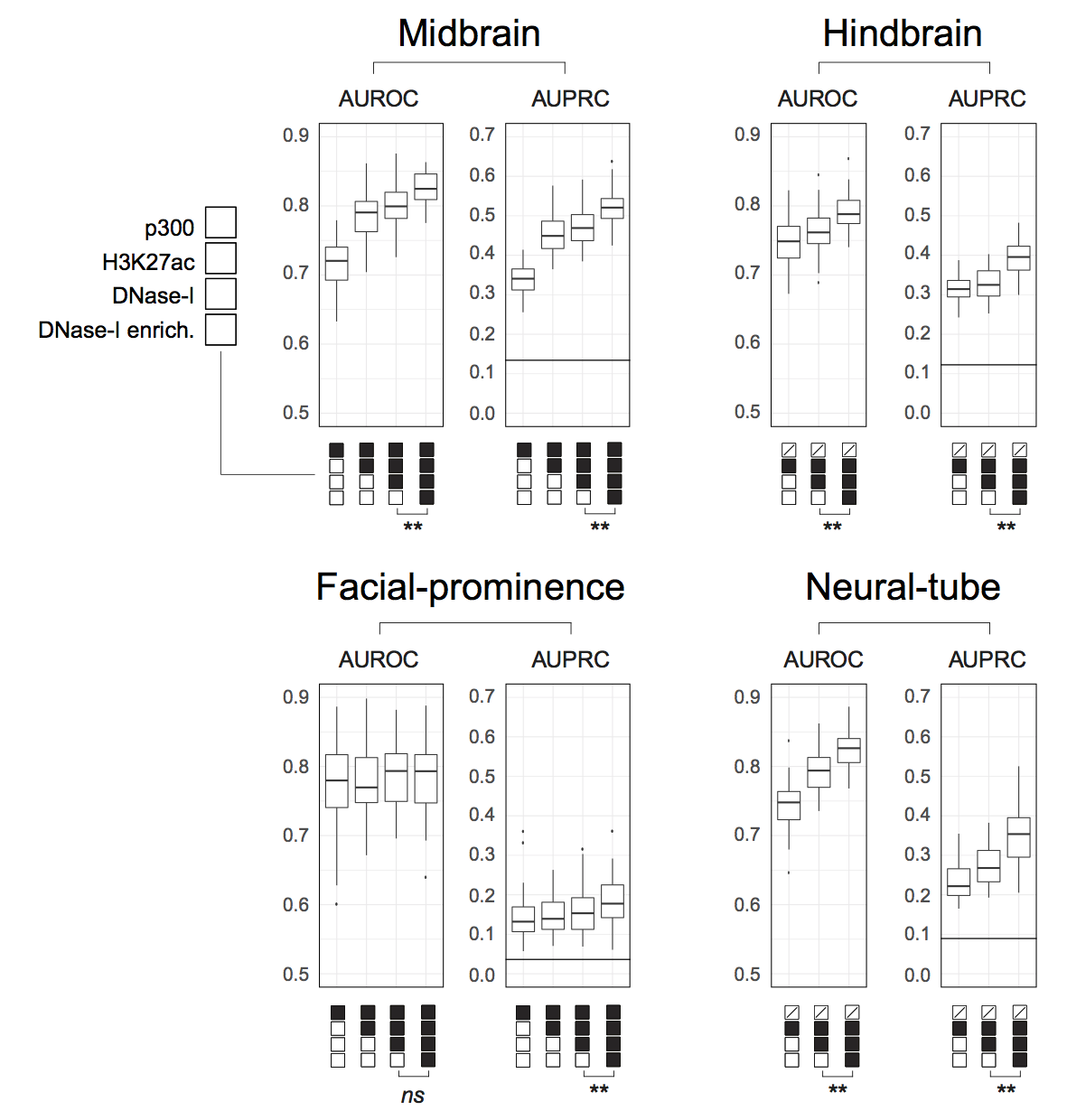

Supplement: S8 Fig — Box plots showing the AUROC and AUPRC estimated by logistic regression on ten rounds of 5-fold cross validation, considering an increasingly larger set of chromatin features (p300 if available, H3K27ac, DNase I accessibily and DNase I enrichment), in E11.5 midbrain, hindbrain, facial prominence and neural tube. The horizontal lines in the AUPRC plots highlight the value expected by chance, given each specific dataset. ** p-value < = 1e-4; ns = not significant, p > 0.05 (one-tailed Wilcoxon signed-rank test). (TIFF) [file pcbi.1005720.s009.tiff]

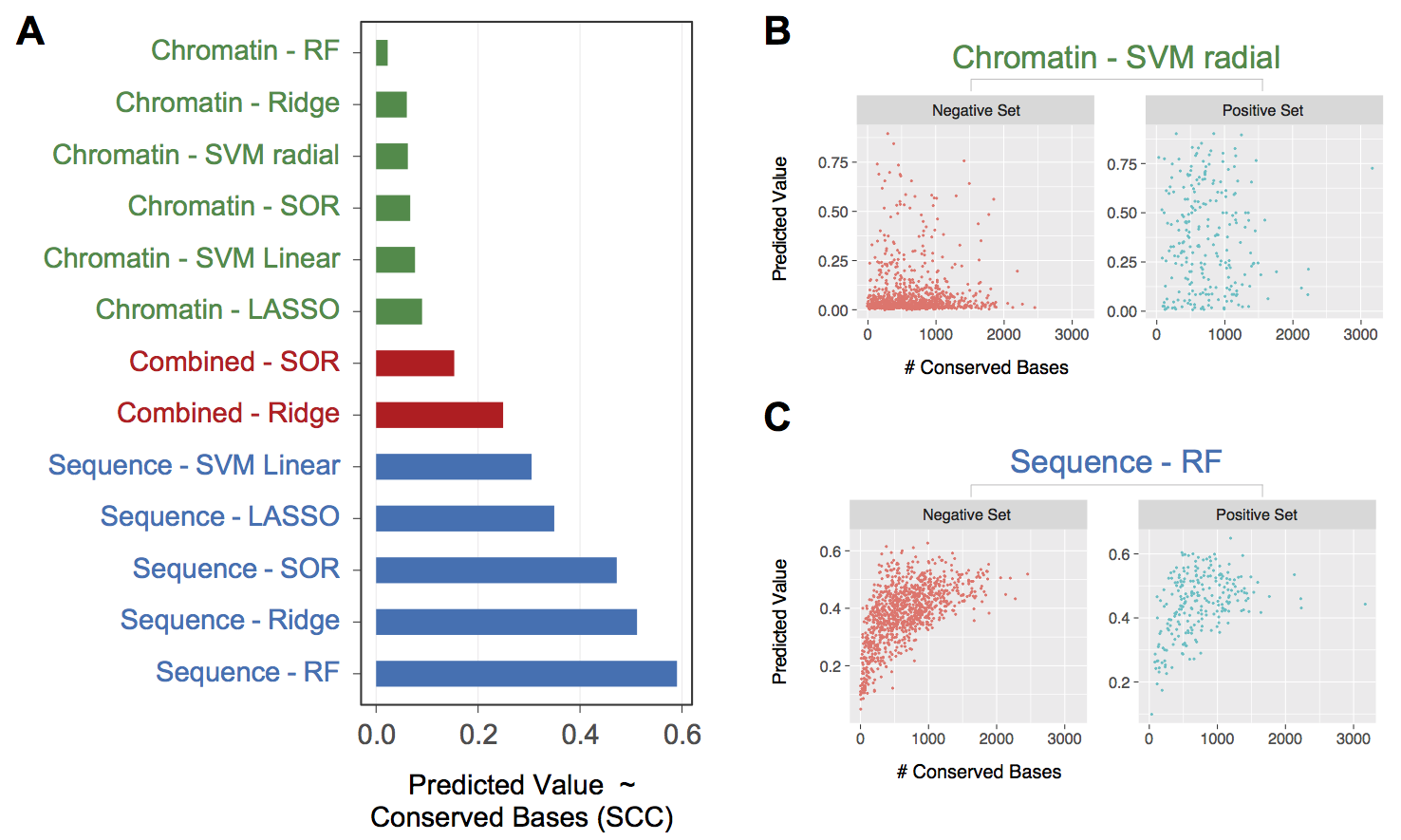

Supplement: S9 Fig — (A) Bar charts showing the Spearman’s Correlation Coefficient (SCC) of the predicted values (on the training set) vs the number of conserved base pairs in each element. (B-C) Scatterplots for negative and positive examples are shown for one chromatin (SVM radial) and one sequence (RF) models. (TIFF) [file pcbi.1005720.s010.tiff]
